# Projecting future labor losses due to heat stress in China under climate change scenarios

**DOI:** 10.1016/j.scib.2023.09.044

**Published:** 2023-11-30

**Authors:** Liangliang Cheng, Kuiying Gu, Liang Zhao, Huibin Wang, John S. Ji, Zhao Liu, Jianbin Huang, Yidan Chen, Xuejie Gao, Ying Xu, Can Wang, Yong Luo, Wenjia Cai, Peng Gong, Wannian Liang, Cunrui Huang

**Affiliations:** a School of Public Health, Sun Yat-sen University, Guangzhou 510080, China; b Vanke School of Public Health, Tsinghua University, Beijing 100084, China; c State Key Laboratory of Numerical Modelling for Atmosphere Sciences and Geophysical Fluid Dynamics, Institute of Atmospheric Physics, Chinese Academy of Sciences, Beijing 100029, China; d School of Linkong Economics and Management, Beijing Institute of Economics and Management, Beijing 100102, China; e Beijing Yanshan Earth Critical Zone National Research Station, University of Chinese Academy of Sciences, Beijing 101408, China; f College of Resources and Environment, University of Chinese Academy of Sciences, Beijing 100190, China; g State Key Joint Laboratory of Environment Simulation and Pollution Control, School of Environment, Tsinghua University, Beijing 100084, China; h College of Earth and Planetary Sciences, University of Chinese Academy of Sciences, Beijing 100084, China; i Climate Change Research Centre, Institute of Atmospheric Physics, Chinese Academy of Sciences, Beijing 100029, China; j National Climate Centre, China Meteorological Administration, Beijing 100081, China; k Department of Earth System Science, Ministry of Education Key Laboratory for Earth System Modeling, Institute for Global Change Studies, Tsinghua University, Beijing 100084, China; l Department of Earth Sciences and Department of Geography, University of Hong Kong, Hong Kong 999077, China; m Institute of Healthy China, Tsinghua University, Beijing 100084, China

**Keywords:** Climate change, Heat stress, Labor, Work hours lost, Occupational health

## Abstract

Climate change is expected to increase occupational heat stress, which will lead to diminished work performance and labor losses worldwide. However, sub-regional analyses remain insufficient, especially for countries with a heterogeneous spatial distribution of working populations, industries and climates. Here, we projected heat-induced labor losses in China, by considering local climate simulations, working population characteristics and developing an exposure–response function suitable for Chinese workers. We showed that the annual heat-induced work hours lost (WHL), compared to the baseline of 21.3 billion hours, will increase by 121.1% (111.2%–131.1%), 10.8% (8.3%–15.3%), and −17.8% (−15.3%–−20.3%) by the end of the century under RCP(Representative Concentration Pathways)8.5, RCP4.5, and RCP2.6, respectively. We observed an approximately linear upward trend of WHL under RCP8.5, despite the decrease in future working population. Notably, WHL will be most prominent in the southern, eastern and central regions, with Guangdong and Henan accounting for a quarter of national total losses; this is largely due to their higher temperature exposure, larger population size, and higher shares of vulnerable population in total employment. In addition, limiting global warming to 1.5 °C would yield substantial gains. Compared to RCP2.6, RCP4.5, and RCP8.5, all provinces can avoid an average of 11.8%, 33.7%, and 53.9% of annual WHL if the 1.5 °C target is achieved, which is equivalent to avoiding 0.1%, 0.6%, and 1.4% of annual GDP losses in China, respectively. This study revealed climate change will exacerbate future labor losses, and adverse impacts can be minimized by adopting stringent mitigation policies coupled with effective adaptation measures. Policymakers in each province should tailor occupation health protection measures to their circumstances.

## 1 Introduction

Climate change affects occupational health and work efficiency through increased intensity and frequency of extreme heat events, especially for workers exposed to high temperatures and humidity [1], [2]. Heat stress in the workplace was first mentioned in the Fourth Assessment Report of the Intergovernmental Panel on Climate Change (IPCC) and was given more attention in the IPCC’s Fifth and Sixth Assessments Reports [3]. Labor loss due to reductions in work rates under climate change is a growing problem around the globe; it has caused over 650 billion working hours of lost labor and a potential cost of $280–$311 billion per year [4], [5], [6], [7]. Thus, a comprehensive understanding of future heat exposure and associated labor loss is needed to inform decision-making and policies that support mitigation or adaptation to climate change.

Based on exposure–response functions (ERFs) between temperature and labor productivity, previous studies have projected heat-related labor losses from both a global and regional scale, and demonstrated that continued warming will lead to the most serious working hour lost in the tropics and midlatitudes during peak months of heat stress [2], [8], [9], [10], [11], [12], [13], [14], [15]. However, studies at the global scale mainly used coarse global climate models (GCMs), adopted a unified ERF overlooking the regional thermal sensitivity, and did not consider the spatial distribution of the working population differences by region or industry, so the results were imprecise and unconvincing [8], [9], [10]. On the other hand, despite some countries’ attempts at making country-level projections, such as the US, South Korea, Japan, Germany, and India [11], [12], [13], [14], [15], sub-national/regional analyses remain insufficient. In particular, countries with a highly heterogeneous spatial distribution of working population, industries, and climates are understudied, and the factors that contribute to the temporal and spatial dynamics of heat-related labor losses in those countries remain unclear.

China, the most populous developing country with complex climate conditions, represents a typical example of assessing heat-related impacts on labor under climate change scenarios [16]. Although several studies have already projected heat-related labor losses in China, their ERFs were simply work/rest ratios in hot weather suggested by experts or health institutions. These suggestions have been criticized since their objective was to minimize the core body temperature to prevent serious illness, but never intended to represent the decline in heat-related labor output [17], [18], [19]. Other studies directly used ERFs from epidemiological surveys from other countries, which cannot truly reflect the heat sensitivity of the Chinese [20]. In addition, most of them conducted analyses in large climate zones and assumed a fixed percentage of the working population in different industries in all regions of the country [17], [18]. These assumptions and coarse analyses limited the ability of previous studies to provide real-world evidence for detailed interventions.

Given that unsustainable development is increasing the health risk for humans, limiting global warming becomes an essential task [21], [22]. The international community reached an agreement at the United Nations Framework Convention on Climate Change 21st Conference of Parties, which set long-term goals to pursue efforts to limit the temperature increase to below 1.5 °C. The 1.5 °C goal may significantly reduce the impacts of climate change. Previous studies have evaluated the benefits for human health by limiting global warming. For instance, tens of thousands of additional deaths will be avoided in China annually under 1.5 °C warming compared with 2 °C warming [23]. However, few studies in China have explored the impact on labor, and such evidence is critical for policymakers to fully understand the importance of ambitious climate targets from an occupational health perspective.

This study is pioneering in assessing future heat-related labor losses in China by utilizing Chinese worker-specific epidemiological functions and considering the spatial distribution of the working population. Specifically, we derived climate data from a regional climate model, and estimated working population based on the dynamic development of future urbanization. We also calibrated the global epidemiological ERF according to Chinese occupational health standards and obtained a function suitable for Chinese workers. On this basis, we revealed the temporal and spatial dynamics of future heat-related labor losses under different climate change scenarios, and explored the drivers of changes in the loss. To be more policy-relevant, we further revealed the health benefits on labor if the 1.5 °C goal is achieved. This study belongs to an initiative of the Lancet Countdown Asia Centre which aims to provide a multi-dimensional projection of future health risks from climate change in China.

## 2 Materials and methods

### 2.1 Estimation of gridded wet bulb globe temperature (WBGT)

The climate data we used were a regional climate model (RegCM4.4) simulation at a spacing of 25 km over the CORDEX-East Asia domain driven by three CMIP5 GCMs, including HadGEM2-ES, MPI-ESM-MR, and NorESM1-M. Using a regional climate model not only provides climate information with higher resolution, but also shows better performance in reproducing the present climate over China [24], [25], [26]. Three different GCMs were used to drive RegCM4.4 to address climate uncertainties, since these GCMs come from different model groups with different structures, roughly cover the range of CMIP5 models’ climate sensitivities, and perform well over East Asia [27], [28] (Supplementary materials online).

Three emission scenarios were used in the climate data, covering the full range of pathways, from RCP2.6 closer to the lower end, RCP4.5 in the middle, to RCP8.5 at the high end of the range. Based on the quantile delta mapping (QDM) method, we derived bias-corrected daily near-surface mean/max air temperature, relative humidity, near-surface wind speed, and surface downwelling shortwave radiation in the baseline (1986–2005) and future periods (2021–2100) under the three RCP scenarios [29]. We used WBGT to assess heat stress for workers in hot environments, as it is an internationally recognized metric in the field of occupational health [30], [31]. We estimated gridded daily indoor WBGT based on temperature and relative humidity, and the calculation of the outdoor WBGT also included the effect of wind speed and radiation [32], [33]. Since future projections of hourly climate variables are not available, we approximately estimated the hourly WBGT based on the daily WBGT using the “4 + 4 + 4” method [8]. With an assumption of working 8 h a day (the legal working time according to Chinese labor law), we estimated gridded hourly indoor and outdoor WBGT during the work time from 1986 to 2100 (detailed meteorological algorithms, the “4 + 4 + 4” method and assumptions in Supplementary materials online).

### 2.2 Projections of gridded working population

Considering China’s possible development patterns and population policies, we used the Shared Socioeconomic Pathway 2 (SSP2) for population projections because it depicts a very likely development scenario for China in the future [20], [34]. We derived future gridded yearly population at 1 km × 1 km resolution under three possible fertility scenarios (low, moderate, and high), based on the global population projection by considering recent fertility-promoting policies implemented in China [34]. The future depopulation in China was also considered in the projection data. In addition, we collected historical population data in China from 1986 to 2005 from the global hybrid gridded demographic datasets [35].

As rapid urbanization is an important factor affecting employment in China, we estimated the gridded working population from 1986 to 2100 based on the dynamic development of future urbanization. The calculation formula is as follows:

(1) $$Pop\_agriculture_{\mathrm{ijk}}=\;{(Total\_pop\;}_{\mathrm{ijk}}\times \;Rural\_rate_{\mathrm{ijk}})\times Agriculture\_rate_{\mathrm{ijk}},$$

(2) $${Pop\_non\_agriculture}_{\mathrm{ijk}}=\;{(Total\_pop\;}_{\mathrm{ijk}}\times \;{Urban\_rate}_{\mathrm{ijk}})\times {\mathrm{No}n_{\mathrm{agricultur}e_{\mathrm{rate}}}}_{\mathrm{ijk}},\;$$

(3) $${Total\_working\_pop}_{\mathrm{ijk}}=\;{Pop\_agriculture}_{\mathrm{ijk}}+{Pop\_non\_agriculture}_{\mathrm{ijk}},\;$$

where i, j, and k denote the year, province, and gird, respectively. ${Total\_pop\;}_{\mathrm{ijk}}$ denotes the gridded total population in each province per year. ${Rural\_rate}_{\mathrm{ijk}}$ and ${Urban\_rate}_{\mathrm{ijk}}$ refer to the proportions of people living in rural or urban areas in each province per year, and they were from the gridded datasets for population and economy under Shared Socioeconomic Pathways in China [36]. ${Agriculture\_rate}_{\mathrm{ijk}}$ and ${Non\_agriculture\_rate}_{\mathrm{ijk}}$ refer to the proportions of the population engaged in agriculture and non-agriculture sectors (including construction, manufacturing, and service) to the total rural or urban population, and they were from *China Statistical Yearbooks*. We assumed that ${Agriculture\_rate}_{\mathrm{ijk}}$ and ${Non\_agriculture\_rate}_{\mathrm{ijk}}$ will remain constant in the future. The total gridded working population from 1986 to 2100 is equal to the sum of the agricultural and non-agricultural populations. We aggregated gridded data to 50 km × 50 km for analysis (more calculations and explanations in Supplementary materials online). There are a total of 34 provincial-level administrative units in China (Fig. S1 online).

Considering that air conditioning (AC) may have protective effects on indoor workers (including manufacturing and service workers), we used AC penetration rates in China to estimate the proportion of indoor workers with access to AC. Specifically, we excluded those indoor workers protected by AC and obtained the truly exposed working population [20] (calculation in Supplementary materials online). Since the AC penetration rate in the workplace was not available, we used the household AC penetration rate instead [20]. The average AC penetration rate in the historical period was obtained from the data on the ownership of AC systems per 100 households from *China Statistical Yearbooks*. The average AC penetration rate has reached nearly 60%, which is consistent with the rate reported by the International Energy Agency (https://www.iea.org/data-and-statistics/charts/percentage-of-households-equiped-with-ac-in-selected-countries-2018). In the study, we assumed that the future AC penetration rate will be constant and estimated the associated labor loss accordingly.

### 2.3 Development of ERFs between WBGT and labor productivity

Although several ERFs between WBGT and labor productivity have been developed in previous studies, the epidemiological ERF developed by Kjellstrom et al. [8] was the most widely used worldwide, as its source data came from epidemiolocal filed surveys rather than empirical data (Fig. S2 online). This function has been prioritized in many studies to estimate heat-induced labor loss worldwide [6], [8], [30], [37].

Since the global widely-used ERF (short for “global ERF”) was not specifically developed for China, we decided to adjust the global ERF according to the Chinese occupational health standard to obtain a function suitable for Chinese workers. The occupational health standard was used as it incorporated heat sensitivity of local workers in hot environments. We adjusted the global ERF using the localized Chinese occupational health standard, which can better reflect heat sensitivity of the Chinese population. In addition, the occupational health standard was issued by the Chinese government with reliable and credible data sources.

We first fitted exposure–response curves between WBGT and labor productivity for different work intensities (200, 300, and 400 W) by using a cumulative distribution function, based on the recommended work/rest ratios from the Chinese occupational health standard (Fig. S3a online) [38]. We estimated the function parameters of the ERF developed by the occupational health standard, and calculated the differences in function parameters of 200 and 400 W compared to 300 W (Table S2 online). Secondly, compared with the ERF developed by the occupational health standard, the ERF developed by epidemiological surveys would give a more reliable estimate. As the exposure–response curve of 300 W in the global ERF was developed by epidemiological surveys, we used the curve of 300 W and the corresponding function parameters in our Chinese epidemiological ERF. To further estimate the exposure–response curves and function parameters of 200 and 400 W, we used the differences in parameters between the three curves in the first step to extrapolate the epidemiological exposure–response curves of 200 and 400 W for Chinese epidemiological ERF. The formula of the adjusted ERF is as follows:

(4) $$\mathrm{Loss}\;fraction=\frac{1}{2}\times \left(1+erf\left(\frac{WBGT-Prod_{mean}}{Prod_{sd}\times \sqrt{2}}\right)\right),$$

where the erf is a cumulative distribution function. ${Prod}_{mean}$ and ${Prod}_{sd}$ are function parameters for working with different intensities of 200, 300, and 400 W (Table S3 and Fig. S3b online). The Lossfraction is the percentage of work time lost to the total work time.

We assumed that agriculture and construction were high-intensity jobs (400 W), manufacturing was a moderate-intensity job (300 W), and service was a low-intensity job (200 W) [39]. Therefore, this function is not only applicable to outdoor workers such as agricultural and construction workers, but also to indoor workers such as manufacturing and service workers. The study covered the entire working population including agriculture, construction, manufacturing, and services in China [6].

### 2.4 Projecting future labor losses due to heat stress

In the study, labor loss is defined as the reduction of hourly work capacity at different levels of work intensity, and is measured as an equivalent number of work hours lost (WHL) [6], [7], [30], [37]. Based on the adjusted ERF, we linked different work intensities with different industries, and used the gridded hourly WBGT to estimate gridded hourly WHL per capita. We multiplied it by the gridded number of working populations to obtain the total WHL. We aggregated the gridded estimates to the provincial and national levels, and showed the WHL in the early (2021–2040, center on 2030), middle (2051–2070, center on 2060), and late (2081–2100, center on 2090) future periods under the different RCP scenarios.

We adopted the factor separation method to estimate drivers contributing to the temporal changes in WHL. As the calculation of WHL was mainly based on future climates and the number of working populations, we considered three drivers, including climate effect, population effect, and their interaction effect [40]. Briefly, we isolated the impacts of population and climate by recalculating WHL when one factor was held constant [41]. The climate effect revealed the contribution of climate factors to the WHL. The population effect revealed the contribution of population factors to the WHL. The interaction effect is the remaining effect after subtracting the climate and population effects from the total WHL (detailed calculation in Supplementary materials online).

To be more policy-relevant, we revealed the health benefits on labor if the 1.5 °C goal is achieved. Since no climate trajectory or scenario directly corresponds to the 1.5 °C goal, we made an approximate calculation. It is calculated that 2021–2040 under RCP2.6 is the time frame for global warming to reach 1.5 °C compared with pre-industrial levels, so the climate data are taken as the 1.5 °C scenario (Supplementary materials online) [42]. By controlling for other factors like population, we only used the WBGT differences between the 1.5 °C scenario and three RCP scenarios in 2051–2070, and then substituted the differences into the ERF and multiplied by the average population in 2051–2070 to obtain WHL. The estimate indicates the net benefits (or avoided WHL) of achieving the 1.5 °C goal in the mid-century. In addition, we used the human capital (HC) method to estimate the economic cost of avoided WHL [2], [5]. Specifically, the economic cost was the product of the avoided WHL and its unit value (Supplementary materials online).

### 2.5 Uncertainty analysis

Several sources of uncertainty were considered in the study, including the uncertainty in climate projections, future population size, different ERFs, daily working hours, and AC penetration rates in the future. The first was the uncertainty in climate projections, and we reported the estimates as ensemble averages under the three GCMs and used 95% CIs. Another uncertainty we have considered was the future population size, and we estimated the WHL under low, moderate, and high fertility rates scenarios in the future. In addition, the ERF we used may have uncertainties, and we compared the estimates based on China’s and global ERFs, respectively.

In China, workers usually work overtime, especially in large and densely populated cities. Most of them do not go to work early but postpone the time of getting off work. Therefore, we conducted the uncertainty analysis of different delayed working hours, considering two common scenarios including a 1-h delay and a 2-h delay in the late evening. Given China’s rapid economic development, there is uncertainty about the future penetration rate of AC [39], [43]. We considered three scenarios of the future average AC penetration rate, increasing by 10%, 20%, and 30% to reach 70%, 80%, and 90% by the end of the century (detailed assumptions in Supplementary materials online).

## 3 Results

**Fig. 1:**
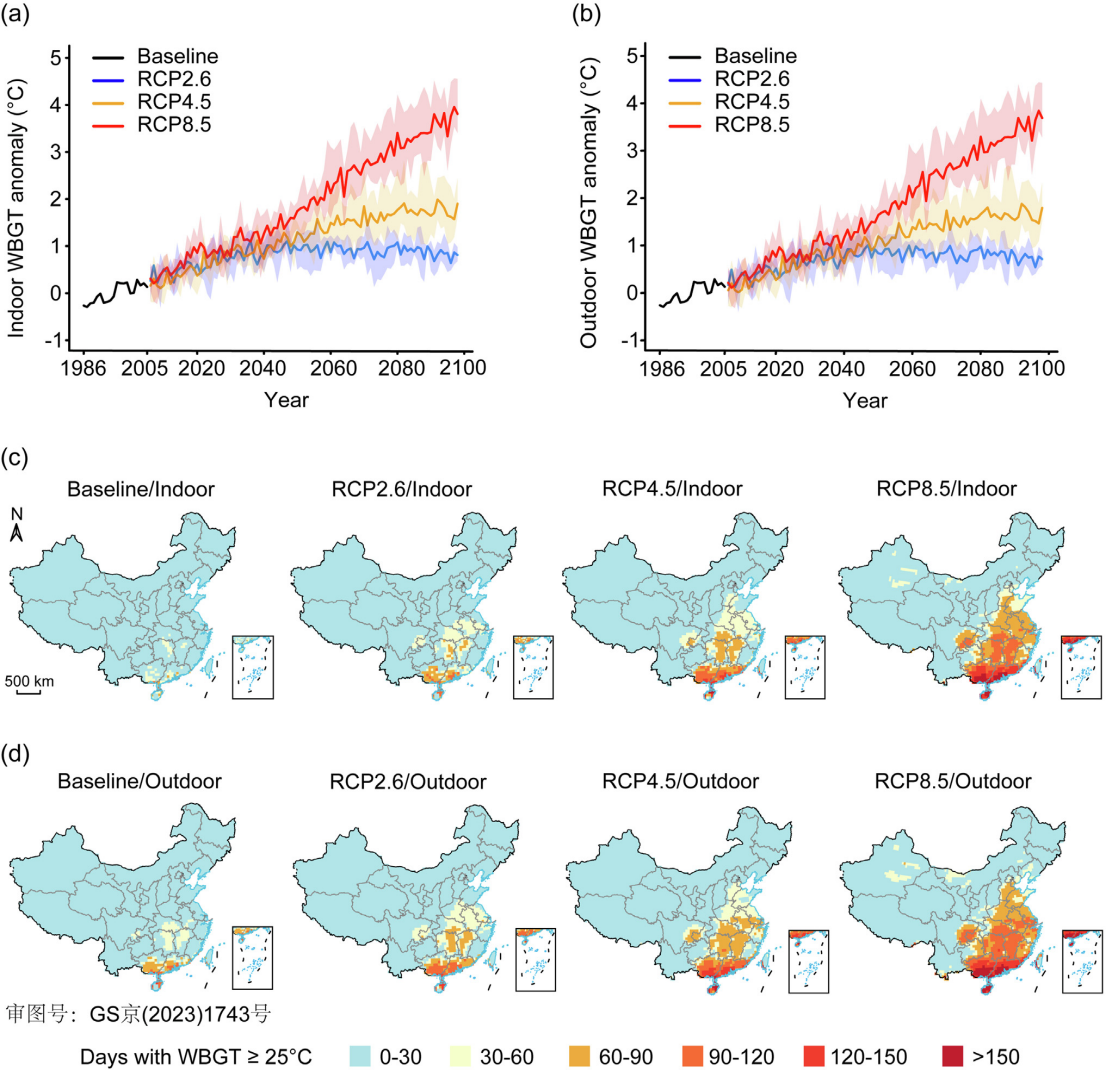
Temporal and spatial trends of projected WBGT in China. (a) Changes of annual indoor WBGT under different climate change scenarios. (b) Changes of annual outdoor WBGT under different climate change scenarios. (c) Spatial distribution of the annual number of days with indoor WBGT exceeding the WBGT threshold (≥25 °C) during the baseline period and in the late century. (d) Spatial distribution of the annual number of days with outdoor WBGT exceeding the WBGT threshold (≥25 °C) during the baseline period and in the late century. Baseline period: 1986–2005. The late-century: 2081–2100. RCP2.6, RCP4.5, and RCP8.5 denote the low, medium, and high emission scenarios, respectively. The shaded areas represent the WBGT range under three GCMs for each year.

Fig. 1 presents the changes in projected WBGT under the three RCP scenarios. Consistent increases are observed under both RCP8.5 and RCP4.5, while an increasing and then flat trend of WBGT is observed under RCP2.6 (Fig. 1a, b and Fig. S4 online). In 2090, the average outdoor WBGT under RCP8.5 and RCP4.5 will increase by 3.4 and 1.6 °C compared with the baseline (1986–2005), respectively. In addition, the southeastern and central regions will see more heat events (Fig. 1c, d). The working population in China will continue to grow in the early part of this century, peaking at around 0.75 billion in 2030, and then show a decline in the second half of this century (Fig. S5 online).

**Fig. 2:**
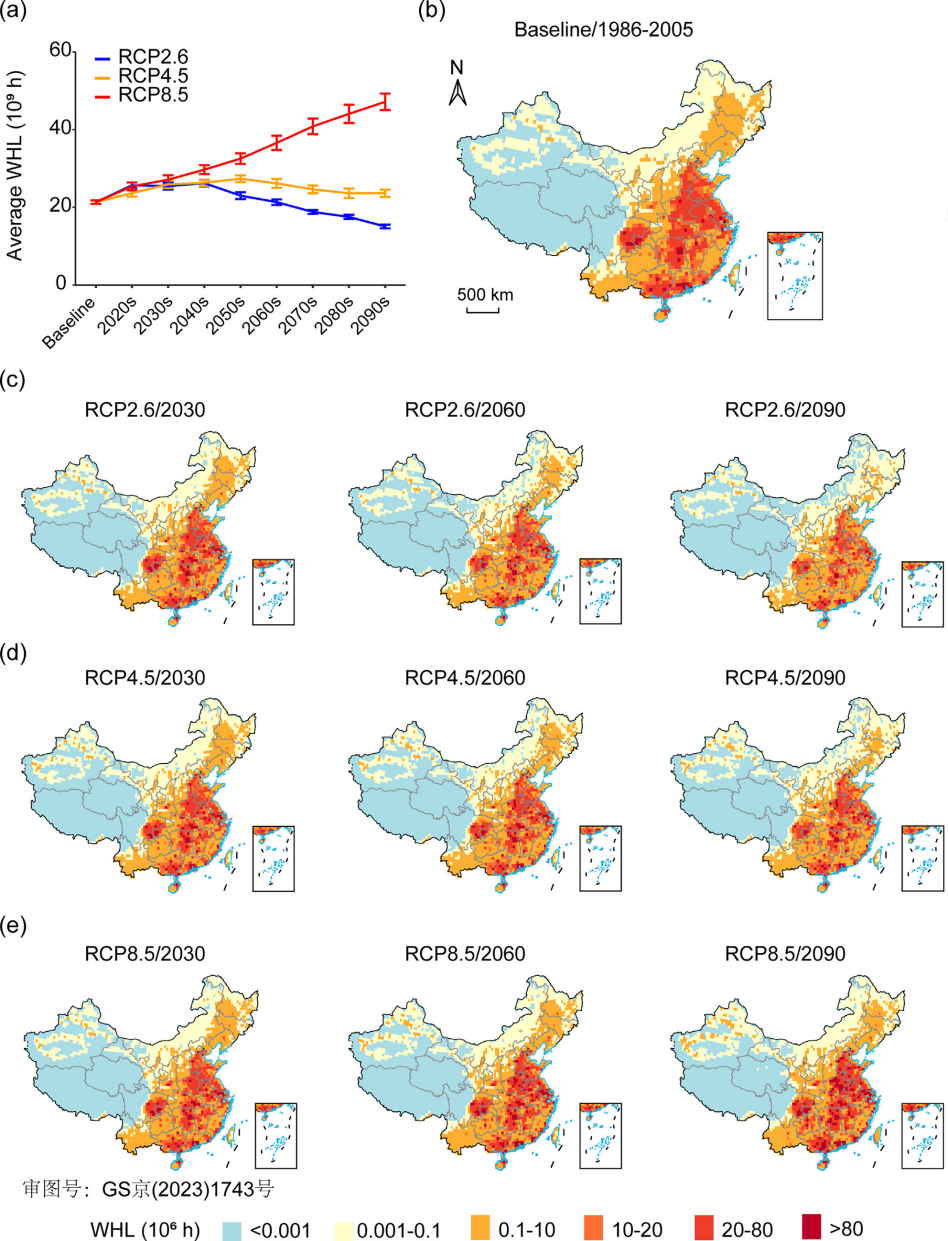
Temporal and spatial dynamics of projected work hours lost (WHL) in China. (a) Future WHL in each decade under the RCP2.6, RCP4.5, and RCP8.5 scenarios. (b) Spatial distribution of the annual average WHL in the baseline period. (c–e) Spatial distribution of the annual average WHL in early, middle, and late periods. 2030 represents the annual average loss from 2021 to 2040 (early future). 2060 represents the annual average loss from 2051 to 2070 (middle future). 2090 represents the annual average loss from 2081 to 2100 (late future). The effort bar denotes the 95% confidence interval.

Based on the most likely moderate fertility scenario, we found that the annual WHL due to heat stress will decrease by 17.8% (15.3%–20.3%) under RCP2.6 at the end of the century, compared to the baseline WHL of 21.3 billion hours (Fig. 2a). However, annual WHL under RCP4.5 and RCP8.5 will significantly increase by 10.8% (8.3%–15.3%) and 121.1% (111.2%–131.1%) at the end of the century compared to the baseline period, respectively. We projected a steep increase in WHL under RCP8.5, while a first increase and then decline present in other RCP scenarios. By 2090, the WHL under RCP8.5 and RCP4.5 will be 32.1 (31.0–34.1) billion hours and 8.5 (7.4–10.5) billion hours higher than that under RCP2.6, which is equivalent to 11 million and 3 million full-time jobs, respectively. From the perspective of WHL per capita, the WHL per capita under RCP8.5 and RCP4.5 will be 96 (92–102) h and 26 (22–31) h higher than that under RCP2.6 in 2090.

**Fig. 3:**
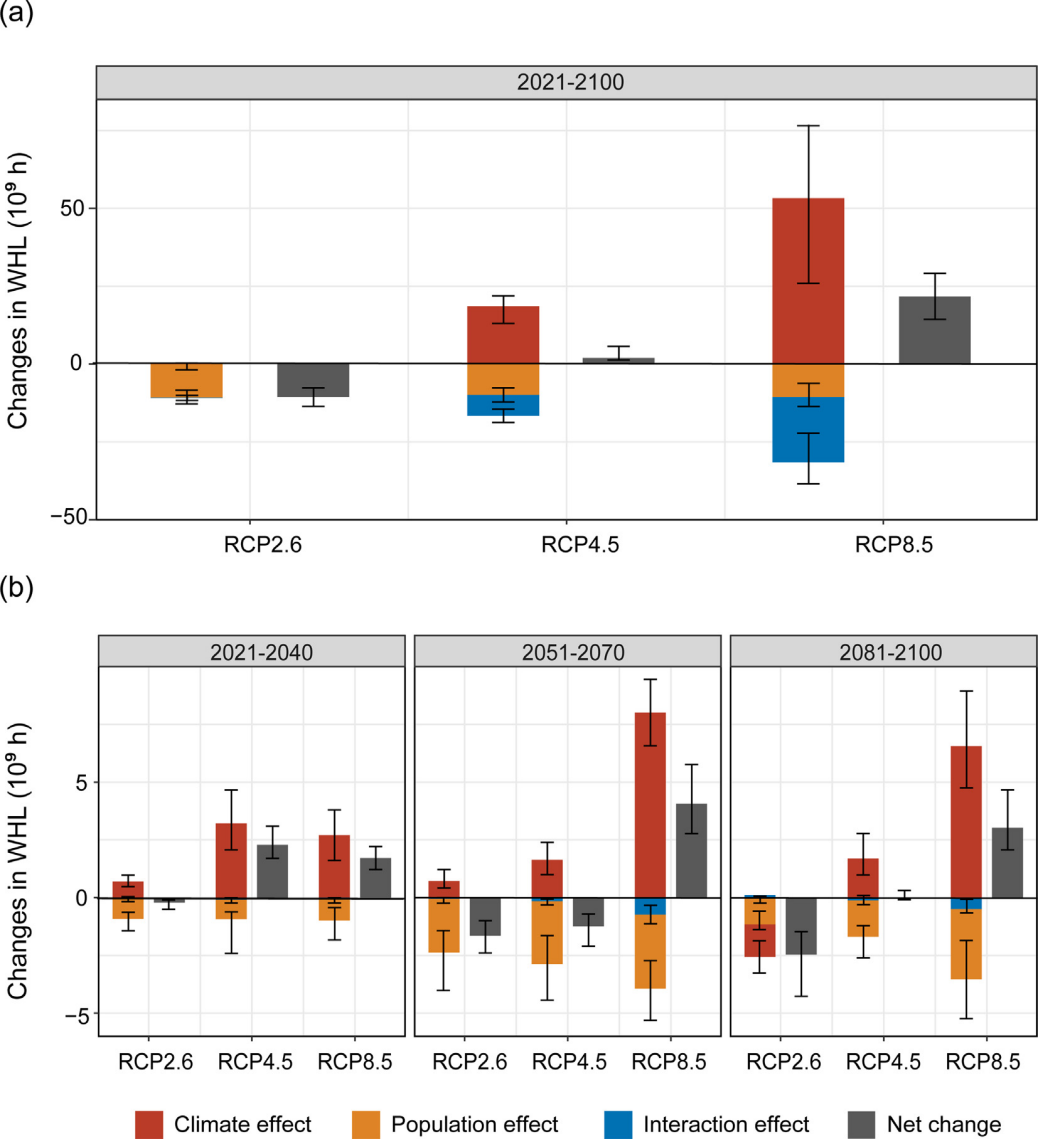
Drivers of future changes in WHL under the three RCP scenarios. (a) Drivers of future changes in WHL under the three RCP scenarios during 2021–2100. The interaction effect is the remaining effect after subtracting the climate and population effects from the total effect. It indicates the impact on labor productivity when WBGT and population are simultaneously changed from the 2020s to 2100s levels. (b) Drivers of future changes in WHL in the early, middle, and late periods under the three RCP scenarios. The effort bar denotes the 95% confidence interval.

In 2021–2100, a net decrease in WHL is observed under RCP2.6, whereas net increases are observed under RCP4.5 and RCP8.5 (Fig. 3a and Table S5 online). The decrease in WHL under RCP2.6 is largely due to a future reduction in the working population, which explains 97% of the total changes. However, under RCP4.5 and RCP8.5, the rising temperature will increase annual WHL by 18.6 (14.2–23.8) billion and 53.3 (30.1–70.7) billion hours in 2021–2100 respectively, which far exceed the decline in WHL due to a reduction in the working population. In addition, we identified the contributing factors in different future periods (Fig. 3b and Table S5 online). There is significant heterogeneity for different periods, but the rising temperature still explains the majority of the WHL increase.

Fig. 2b–e shows the differences in WHL among regions under the three RCP scenarios. The WHL will change across regions, with effects spreading along the south, east and central China. During 2021–2100, the average WHL in the southern, eastern and central regions will be 4.3 (3.6–5.3), 4.0 (3.6–4.7), and 3.8 (3.5–4.4) times that in other regions under RCP2.6, RCP4.5, and RCP8.5, respectively. Although the difference in the spatial distribution of WHL under three scenarios is not significant due to rising temperature and population in the early future, it can be observed that WHL in southern, eastern, and central regions will be more severe under RCP8.5 in 2090, compared with RCP2.6 (Fig. 2c–e).

In addition, there will be great differences in WHL among 34 provincial-level administrative units in China (Table 1). We projected that one-third of all provinces will account for 80% of the country’s losses during 2021–2100, most of which are in southern, eastern, and central regions. Notably, the top two provinces with the largest losses in China are not geographically adjacent, one being Guangdong Province in southern China with an annual WHL of 4.7 (3.5–6.2) billion hours under RCP8.5 during 2021–2100, followed by Henan Province in central China with a corresponding WHL of 3.7 (2.9–4.6) billion hours. These two provinces will have the largest WHL, accounting for a quarter of national total losses. We also quantify WHL by provinces in different future stages, and the rankings of WHL do not vary significantly (Tables S6–S8 online). In addition, the WHL of outdoor workers will be much higher than that of indoor workers in most provinces (Table S9 online).

**Table 1:** Future WHL under the three RCP scenarios by province in China^a^.

Province	RCP2.6		RCP4.5		RCP8.5	
	Rank	WHL (proportion)	Rank	WHL (proportion)	Rank	WHL (proportion)
Guangdong	1	2.8 (13.1%)	1	3.3 (12.9%)	1	4.7 (13.4%)
Henan	2	2.2 (10.1%)	2	2.6 (10.2%)	2	3.7 (10.3%)
Guangxi	3	1.9 (8.8%)	4	2.2 (8.7%)	4	2.9 (8.2%)
Hunan	4	1.9 (8.7%)	3	2.2 (8.7%)	3	3.1 (8.7%)
Jiangsu	5	1.7 (7.7%)	5	1.9 (7.5%)	5	2.6 (7.2%)
Hubei	6	1.7 (7.6%)	6	1.8 (7.3%)	6	2.4 (6.8%)
Sichuan	7	1.6 (7.1%)	7	1.8 (7.0%)	7	2.4 (6.7%)
Anhui	8	1.4 (6.3%)	8	1.6 (6.2%)	8	2.1 (6.0%)
Jiangxi	9	1.2 (5.7%)	9	1.4 (5.7%)	9	1.9 (5.5%)
Shandong	10	1.1 (4.9%)	10	1.3 (5.0%)	10	1.8 (5.0%)
Chongqing	11	0.9 (4.3%)	11	1.1 (4.3%)	11	1.5 (4.2%)
Hebei	12	0.8 (3.5%)	13	0.9 (3.5%)	12	1.3 (3.6%)
Zhejiang	13	0.7 (3.4%)	12	0.9 (3.6%)	13	1.2 (3.5%)
Fujian	14	0.4 (2.0%)	14	0.5 (2.1%)	14	0.8 (2.2%)
Guizhou	15	0.3 (1.3%)	15	0.4 (1.5%)	15	0.7 (2.0%)
Shaanxi	16	0.2 (1.0%)	16	0.3 (1.0%)	16	0.4 (1.1%)
Hainan	17	0.2 (0.9%)	17	0.3 (1.0%)	17	0.4 (1.1%)
Shanghai	18	0.2 (0.7%)	18	0.2 (0.7%)	18	0.3 (0.7%)
Tianjin	19	0.1 (0.5%)	19	0.1 (0.5%)	21	0.2 (0.5%)
Beijing	20	0.1 (0.5%)	20	0.1 (0.5%)	19	0.2 (0.6%)
Liaoning	21	0.1 (0.3%)	21	0.1 (0.4%)	22	0.1 (0.4%)
Shanxi	22	0.1 (0.3%)	22	0.1 (0.3%)	23	0.1 (0.4%)
Yunnan	23	0.1 (0.3%)	23	0.1 (0.3%)	20	0.2 (0.6%)
Heilongjiang	24	0.0 (0.2%)	24	0.1 (0.2%)	24	0.1 (0.2%)
Taiwan	25	0.0 (0.2%)	25	0.0 (0.2%)	25	0.1 (0.2%)
Jilin	26	0.0 (0.2%)	26	0.0 (0.2%)	26	0.1 (0.2%)
Nei Mongol	27	0.0 (0.1%)	27	0.0 (0.1%)	27	0.0 (0.1%)
Hongkong	28	0.0 (0.1%)	28	0.0 (0.1%)	28	0.0 (0.1%)
Gansu	29	0.0 (0.0%)	30	0.0 (0.1%)	30	0.0 (0.1%)
Xinjiang	30	0.0 (0.0%)	29	0.0 (0.1%)	29	0.0 (0.1%)
Ningxia	31	0.0 (0.0%)	31	0.0 (0.0%)	31	0.0 (0.1%)
Macau	32	0.0 (0.0%)	32	0.0 (0.0%)	32	0.0 (0.0%)
Xizang	33	0.0 (0.0%)	33	0.0 (0.0%)	33	0.0 (0.0%)
Qinghai	34	0.0 (0.0%)	34	0.0 (0.0%)	34	0.0 (0.0%)
Total		21.7 (100%)		25.2 (100%)		35.4 (100%)

^a^ The unit of WHL is billion hours; the proportion represents the percentage of WHL to total WHL; 0.0 in and out of brackets represents the value is less than 0.001, but not equal to 0.

**Fig. 4:**
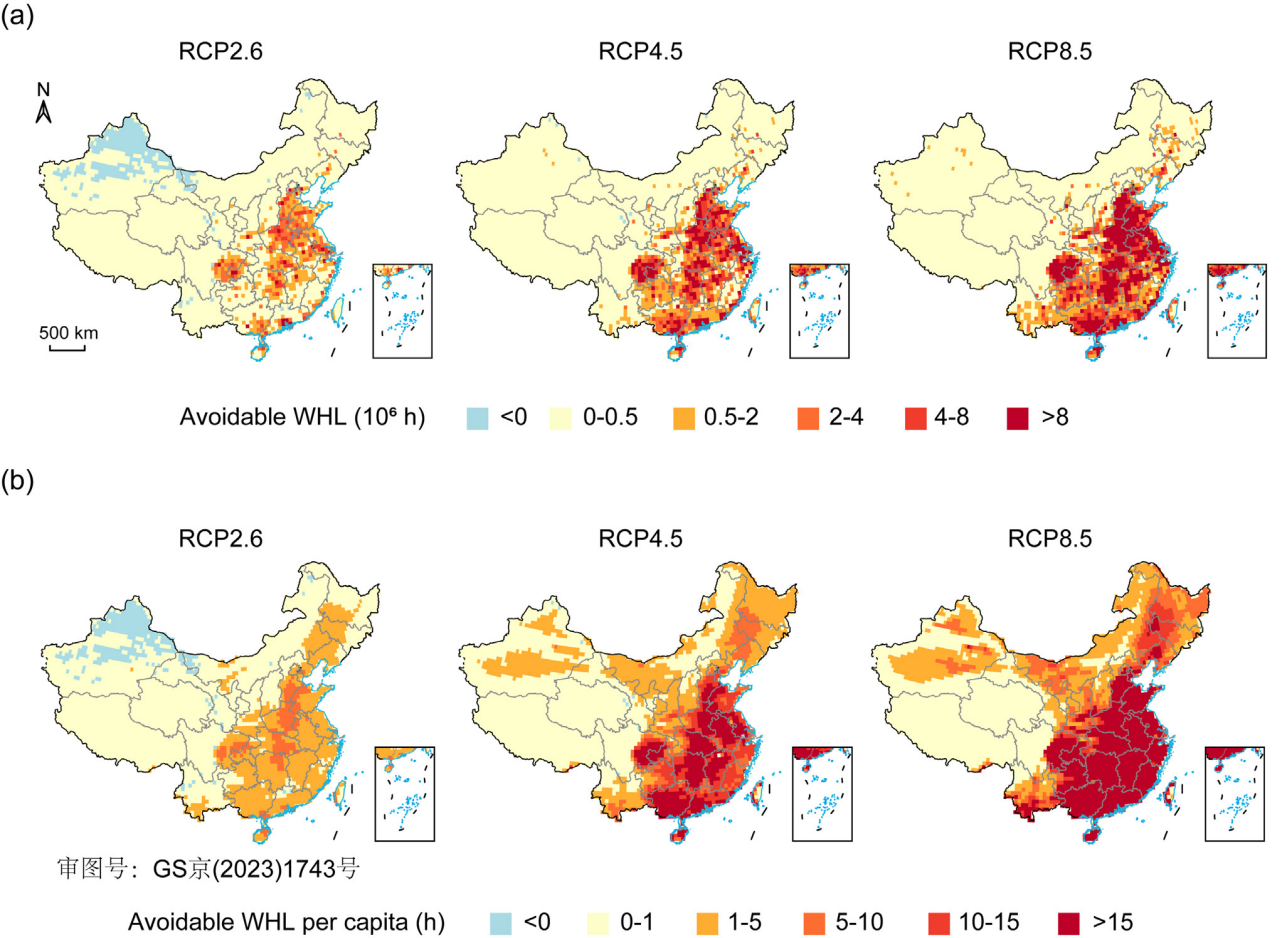
The avoidable WHL under the 1.5 °C scenario compared with three RCP scenarios in mid-century. (a) Total avoidable WHL if achieving the 1.5 °C goal in mid-century (2051–2070). (b) Avoidable WHL per capita if achieving the 1.5 °C goal in mid-century (2051–2070).

Fig. 4 shows the avoided WHL in different regions if achieving the 1.5 °C goal by mid-century. Compared with the RCP2.6, RCP4.5, and RCP8.5 scenarios, the annual WHL in 2060 could be avoided by 1.5 (1.1–3.1), 6.1 (5.3–7.7), and 14.8 (12.5–18.1) billion hours if achieving the 1.5 °C target, respectively (Fig. 4a). In other words, the annual WHL per capita in 2060 could be avoided by 3 (2–7), 13 (12–17), and 32 (27–39) h if achieving the 1.5 °C target, respectively (Fig. 4b). All provinces can avoid an average of 11.8%, 33.7%, and 53.9% of WHL if achieving the 1.5 °C target, compared with the RCP2.6, RCP4.5, and RCP8.5 scenarios. The largest avoidable WHL is concentrated in the southeast and central provinces in China, which are also the regions with the greatest labor losses under the three RCP scenarios. For instance, Guangdong and Henan provinces can avoid the largest WHL by 2.1 (1.3–3.4) and 1.3 (1.1–2.6) billion hours respectively, compared with the WHL under RCP8.5.

In addition, we found that compared with the RCP2.6, RCP4.5, and RCP8.5 scenarios, the economic costs of annual avoided WHL are 25.7, 100.9, and 237.2 billion USD if achieving the 1.5 °C target, respectively. This is equivalent to avoiding 0.1%, 0.6%, and 1.4% of annual national GDP losses in 2060, respectively. Table S10 (online) shows the avoided GDP losses in each province. For instance, the economic costs of annual avoided WHL in Guangdong and Henan provinces will be 30.9 billion and 22.0 billion USD if achieving the 1.5 °C target, compared with the losses under RCP8.5.

In terms of uncertainty in different ERFs, the annual WHL estimated by the global ERF will be 4.5%, 3.4%, and 1.6% higher than that of the Chinese ERF under RCP2.6, RCP4.5, and RCP8.5 at the end of the century, respectively (Fig. S6 online). Compared with the assumption of working 8 h a day, the annual WHL of working 9 h will increase by 0.4%, 1.1%, and 2.5% under RCP2.6, RCP4.5, and RCP8.5 scenarios at the end of the century, respectively. The annual WHL of working 10 h a day will increase by 0.9%, 2.2%, and 4.8% under RCP2.6, RCP4.5, and RCP8.5 scenarios at the end of the century, respectively (Fig. S7 online).

In addition, the annual WHL under the high and low fertility scenarios will be 9.3% higher and 14.3% lower than the middle fertility scenario for RCP8.5 at the end of the century, respectively. Although there are corresponding increases and decreases in WHL in other RCP scenarios, the overall trend remains the same (Fig. S8 online). In terms of uncertainty in future AC penetration rate, we found if the future AC penetration rate will increase by 10% compared to the current rate, the annual WHL will reduce by 6.7%, 7.3%, and 8.3% under RCP2.6, RCP4.5, and RCP8.5 at the end of the century, respectively. If the future AC penetration rate increases by 30% compared to the current, the annual will reduce by 20.0%, 21.9%, and 24.8% under RCP2.6, RCP4.5, and RCP8.5 at the end of the century, respectively (Fig. S9 online).

## 4 Discussion and conclusion

In this modeling study, we estimated future heat-related labor losses in China based on a regional climate model, considering the spatial distribution of the working population and epidemiological function suitable for Chinese workers. The innovation in our study is we calibrated the global epidemiological ERF according to the Chinese occupational health standard, and then developed a function that provides higher precision estimates for China. Based on the reliable climate and working population projections, as well as the calibrated function, we provided a comprehensive and in-depth estimate to date of heat-induced labor losses in China. We not only assessed future labor loss at the provincial level for the first time, but also explored the drivers of changes in the loss. For a better understanding, we revealed the occupational health benefits of achieving the 1.5 °C goal in the largest developing country.

Consistent with previous studies, we observed a large increase in heat-induced labor losses under the medium and high emission scenarios [8], [18], [20], [44], [45]. A study on a global scale found that labor losses under RCP6.0 will be 2.2 times that under RCP2.6 in China, and country-level analyses showed that labor losses will increase by 40% for heavy work in the late century under RCP8.5 [8], [17]. However, as these studies adopted inappropriate ERF and used oversimplified population assumptions, their estimates were largely biased. In contrast, based on the reliable ERF and working population projections, we revealed that the labor losses from heat exposure will double from current levels under RCP8.5 in the late century, which is much higher than previous estimates [17]. In addition, we explored the drivers of changes and found that rising temperature will dominate the increase in labor losses under the medium and high scenarios, even with a reduced working population. Our findings will alert policymakers of the severity of future heat-related impacts on labor, especially as the impacts could jeopardize health and economic development.

Another contribution of our study is we estimated the future labor impacts at the provincial level. Although previous studies by coarse-spatial analysis have also found that southeastern and central China will be more vulnerable due to higher temperatures and larger exposed populations in these regions, our detailed provincial estimate shed light on other important factors leading to great labor losses [17], [18], [20]. China’s economic boom has drawn rural workers to cities in search of higher incomes, and the scale of migration presents a constantly increasing trend [46]. The economically-developed provinces (e.g., Guangdong in south China) will still have relatively high heat-related labor losses due to a large migrant population, as workers from less developed regions such as the west/northeast migrate to developed provinces. In addition, the industrial structure can have a huge impact on labor. China’s industrial structure is constantly improving, shifting away from agricultural reliance to manufacturing and services. However, some economically underdeveloped provinces (e.g., Henan in central China) will suffer huge heat-related labor losses, which is largely due to their high shares of agricultural workers in total employment, as agriculture is the industry most vulnerable to heat [47], [48]. Our results indicate that China should develop specific interventions in each province, anticipating future climate mitigation, workers migration and industrial structure reform.

We found that a great amount of heat-related labor losses can be avoided by achieving the 1.5 °C goal, and this is the first study to reveal the benefits of this ambitious climate targets on China’s labor sector. Although achieving the goal will not be easy, especially with the raging COVID-19 pandemic, countries should strengthen their ambitions on strict mitigation policies by considering their co-benefits for human health and work capacity in the long run [49], [50], [51], [52]. Previous economic studies have revealed that over 40% of mitigation costs worldwide could be offset by the benefit of reducing adverse heat-induced impacts on labor [39], [53]. In addition, we found that achieving the 1.5 °C goal does not mean the total elimination of labor losses; therefore, adaptation measures are important for reducing occupational heat exposure. Our previous research has found that the Chinese government has issued the Administrative Measures on Heatstroke Prevention to combat the occupational health impacts of extreme heat, and the risk of work-related injuries could decrease by 13% after implementing the policy, which supports the importance of adaptation strategies in protecting future occupational health and associated workability [54].

The relationship between diurnal WBGT and WHL is non-linear [2]. Since the WBGT is much higher during the midday compared to the morning or evening, hourly WHL tends to increase and then decrease during the daytime. Our study also found similar nonlinear characteristics. To avoid excessive heat exposure at midday, one possible measure is to place moving labor from midday to cooler hours [55], [56]. However, the protective effect of the measure will diminish in the future, since WBGT in the early morning will continue to rise to unsafe levels for work under climate change [2]. Therefore, more alternative adaptation measures should be developed to keep workers safe and reduce heat-related labor loss.

There are many sources of uncertainty when conducting modelling studies, especially in terms of climate, population characteristics and exposure–response relationships. In this research, we not only considered the uncertainty in climate models, but also considered different future population sizes, daily working hours, exposure–response functions, and future AC penetration rate. There are no significant differences in labor losses between different lengths of daily working hours, as the time point of overtime is usually in the early evening, and the temperature is not as high as noon. In addition, we adjusted the global ERF to Chinese occupational health standards to obtain a new function suitable for China, and found that the labor losses by the Chinese ERF are slightly lower than that by the global ERF. As part of the epidemiological evidence for the global ERF comes from tropical countries, it is reasonable for us to obtain a lower estimation [8].

Modelling studies are prone to limitations by study design. First, since RCM can better simulate regional climate characteristics, we used climate simulations from RegCM4.4. However, it is difficult to address all possible uncertainties because we used only one RCM driven by three GCMs. In the future, we should strengthen collaboration with meteorologists and use multi-GCM/RCM ensembles, especially the latest CMIP6 models under SSPs-RCPs forcings, to better characterize uncertainties in the climate projections. Second, although we estimated the future occupational population based on the dynamic development of future urbanization, future employment can also be affected by other factors like disease outbreaks, natural disasters, global competition and technological advances. In the future, researchers should strengthen the reliable prediction of China’s employment rates, which is important for improving the robustness of the estimate. Thirdly, although the prescribed method to measure WBGT requires special instruments, we had to estimate WBGT based on climate model data because instruments are not commonly used at weather stations. Previous studies suggested that WBGT from climate model data may be biased due to using multiple complex algorithms, and the calculation of WBGT has many assumptions, for instance, workers need to wear light clothing. In addition, the wind speed at all indoor workplaces is assumed to be 1 m/s, which may be an oversimplification of the real situation [57], [58]. These limitations imply that the calculation of WBGT from climate model data should be treated with caution, and more heat stress indexes should be developed and compared.

In conclusion, this study revealed that climate change will exacerbate heat-related labor losses in China, even with a reduced working population in the future. The southern, eastern, and central provinces of China are the most vulnerable, and policymakers in each province should tailor occupation health protection measures to their own circumstances. Actions taken today will determine the extent of future losses, and we should be aware that adopting stringent mitigation policies coupled with effective adaptation measures can minimize the impact on labor. The evidence in this study has a pertinent role in providing evidence for policies of climate change adaptation and mitigation and may be extrapolated for working populations in other developing countries.

## Conflict of interest

The authors declare that they have no conflict of interest.
