# Supplementary material for: Projecting future labor losses due to heat stress in China under climate change scenarios
Source: Sci Bull (Beijing). 2023 Nov 30;68(22):2827–37. doi: 10.1016/j.scib.2023.09.044 (PMC10694465; doi:10.1016/j.scib.2023.09.044)
Supplement: Supplementary data 1 [file mmc1.docx]

**Supplementary Information**

**Projecting future labor losses due to heat stress in China under climate change scenarios**

**Supplementary Methods**

1. **Estimation of gridded Wet Bulb Globe Temperature (WBGT)**

**1.1 Description of climate projection data**

In general, RCM simulations are the outputs of dynamic downscaling of GCM. Since the regional climate model (RCM) can downscale coarse resolution GCM to produce high-resolution grids of climate variables, and perform better in reproducing the present-day climate over regions, we adopted climate data from RCM simulations. In the study, we used data from RegCM4.4, as it has been shown to have good performance in reproducing the current climate in China with complex terrain [1-3].

Unlike studies that use a large number of GCMs simulations for analysis, several suitable GCMs must be selected to drive the RCM when performing RCM simulations based on the CORDEX-CORE framework [4]. In the data we used, the RegCM4.4 is driven by three specific GCMs: NorESM1-M, MPI-ESM-MR, and HadGEM2-ES. The three specific GCMs were chosen for the RCM because (1) they have high, medium, and low ECSs (equilibrium climate sensitivity) within the CMIP5 ensemble [5]. (2) they perform well in East Asia [1, 2]. In the reference of Chen et al. (2019) [6], the ECS (equilibrium climate sensitivity) of them are 2.80ºC (NorESM1-M), 3.47ºC (MPI-ESM-MR), and 4.63ºC (HadGEM2-ES), respectively, ranging from low to high-end model climate sensitivities.

Although authors have reported simulations using other RCMs such as WRF or PRECIS, the differences in selecting and driving suitable GCMs for each RCM, model domain, resolution, and emission pathways make it difficult to be included in a large ensemble to address all the possible uncertainties. In addition, CMIP6-RCM data is not easily accessible to public health researchers, as it requires specialized meteorological processing, considerable computational effort, and close collaboration. Therefore, CMIP6-RCM data is not yet widely used by the public health community. To date, our team does not have climate data for CMIP6-RCM simulations. In the future, we should strengthen collaboration with meteorologists and use multi-GCM/RCM ensembles, especially the latest CMIP6 models under SSPs-RCPs forcings, to better characterize uncertainties in the climate projections.

**1.2 Climate data under the 1.5℃ scenario**

Following the definition of ‘1.5℃ relative to pre-industrial levels’ in SR1.5, we calculated the rise in global average temperature (combined land surface air and sea surface temperatures) relative to 1850-1900 for CMIP5 models MPI-ESM-MR and NorESM1-M, and 1860-1900 for HadGEM2-ES [7]. The timing of ‘1.5℃’ is established by a 30-year running average of the global average temperature increase that exceeds 1.5℃. The global temperature increase reaching ‘1.5℃’ is projected to occur in 2030 under the RCP2.6 scenario in HadGEM2-ES and MPI-ESM-MR. The temperature values in 2021-2040 are used to represent the climate if global warming reaches 1.5 ℃. Under RCP2.6, the global average temperature increase in the 21st century will be less than 1.5 ℃ under NorESM1-M.

**1.3 Calculation of hourly WBGT**

We adopted the method from Lemke et al. for calculating indoor WBGT, and the method from Liljegren et al. for calculating outdoor WBGT, as these two methods have been proved to be the most appropriate methods [8-10]. The calculation formulas are:

$$\mathrm{WBGT}_{indoor}=0.67 \times Tpwb+0.33\times Ta$$

$${WBGT}_{outdoor}=0.7\times Tnwb+0.2\times Tg+0.1\times Ta$$

where $Tpwb$ is the psychrometric wet bulb temperature, $Ta$ is the ambient temperature, $Tnwb$ is the natural wet bulb temperature and $Tg$ is the globe temperature. $Tpwb$ is calculated from the ambient temperature and dew point temperature by iterations, and the dew point temperature is estimated based on the ambient temperature and relative humidity. $Tnwb$ is a combination of the ambient temperature and relative humidity, but it is also influenced by radiation and wind speed. The calculation of Tg includes both the direct and diffuse components of sunlight, and it is calculated based on the radiation by iterative processes. More details on the calculation of $Tpwb$, $Tnwb$ and $Tg$ can be obtained from previous meteorological literature [8, 9]. Based on meteorological algorithms, gridded daily indoor and outdoor WBGT were estimated.

Since hourly climate data was not available, we used the ‘4+4+4’ method for approximate estimation [10]. The method assumed that 4 hours of a 12- hours daylight day were close to WBGTmax, 4 hours were close to WBGTmean (early morning and early evening), and the remaining 4 hours were assumed to be halfway between WBGTmean and WBGTmax (labeled WBGThalf). According to China’s Labour Law, working 8 hours a day is the legal working time [11]. On this basis, we assumed that a worker works 8 hours a day with 2 hours at WBGTmean, 2 hours at WBGTmax, and 4 hours at WBGThalf as in previous studies [12].

1. **Projections of gridded working population**

**2.1 Estimating future total and working population**

We applied three population scenarios under the shared socioeconomic pathways (SSPs) during 2021-2040 (2030s), 2051-2070 (2060s), 2081-2100 (2090s), including SSP2-S1 (low fertility rate), SSP2-S2 (medium fertility rate), SSP2-S3 (high fertility rate). SSP2 was considered as the scenario closet to the developing trend of Chinese population size, and the gridded population at 1km×1km resolution was from the previous work, in which the future yearly Chinese population was extracted and re-analyzed based on the global population projection by considering recently implemented fertility promoting policies in China [13]. The corresponding geographical locations of each province are shown in Figure S1.

One of the most important factors affecting the employment rate is China's rapid urbanization. Over the past few decades, a large proportion of the population originally living in rural areas has migrated to cities, resulting in a dramatic change in the employment rates of different sectors. For example, there has been a significant decline in the employment rate in agriculture and an increase in the employment rate in other sectors such as services. In addition, the Chinese government has taken several measures to increase the level of urbanization in the future. Therefore, we project the future working population based on the dynamics of the future urbanization level. The calculation formula is as follows.

${Pop\_agriculture}_{ijk}= {(Total\_pop}_{ijk}\times{Rural\_rate}_{ijk})\times$ ${Agriculture\_rate}_{ijk}$

$${Pop\_non\_agriculture}_{ijk}= {(Total\_pop}_{ijk}\times{Urban\_rate}_{ijk})\times{Non\_agriculture\_rate}_{ijk}$$

$${Total\_working \_pop}_{ijk}= {Pop\_agriculture}_{ijk}+ {Pop\_non\_agriculture}_{ijk}$$

Here $i$, $j$, and $k$ denote the year, province and gird respectively. ${Total\_pop}_{ijk}$ denotes the gridded total population in each province in each year. ${Rural\_rate}_{ijk}$ and ${Urban\_rate}_{ijk}$refer to the proportions of people living in rural or urban areas in each province per year. ${Rural\_rate}_{ijk}$ and ${Urban\_rate}_{ijk}$ from 2021 to 2100 were from the gridded datasets for population and economy under Shared Socioeconomic Pathways in China [14]. The datasets were developed using the Population-Development-Environment (PDE) model and the Cobb-Douglas production model with consideration for localized population and economic parameters (<https://doi.org/10.57760/sciencedb.01683>). The projection methodology and data have been peer-reviewed, and are reliable forecasts of China's urbanization trends [15, 16].

${Agriculture\_rate}_{ijk}$ refers to the proportion of the population engaged in agriculture to the total rural population. ${Non\_agriculture\_rate}_{ijk}$ refers to the proportion of the population engaged in other sectors (including construction, manufacturing and service) to the total urban population. ${Agriculture\_rate}_{ijk}$ and ${Non\_agriculture\_rate}_{ijk}$ were derived from the China Statistical Yearbooks. As the two rates were relatively stable in the past 10 years, we assumed that they would remain constant in the future. The total working population in the future is equal to the sum of the agricultural and non-agricultural populations.


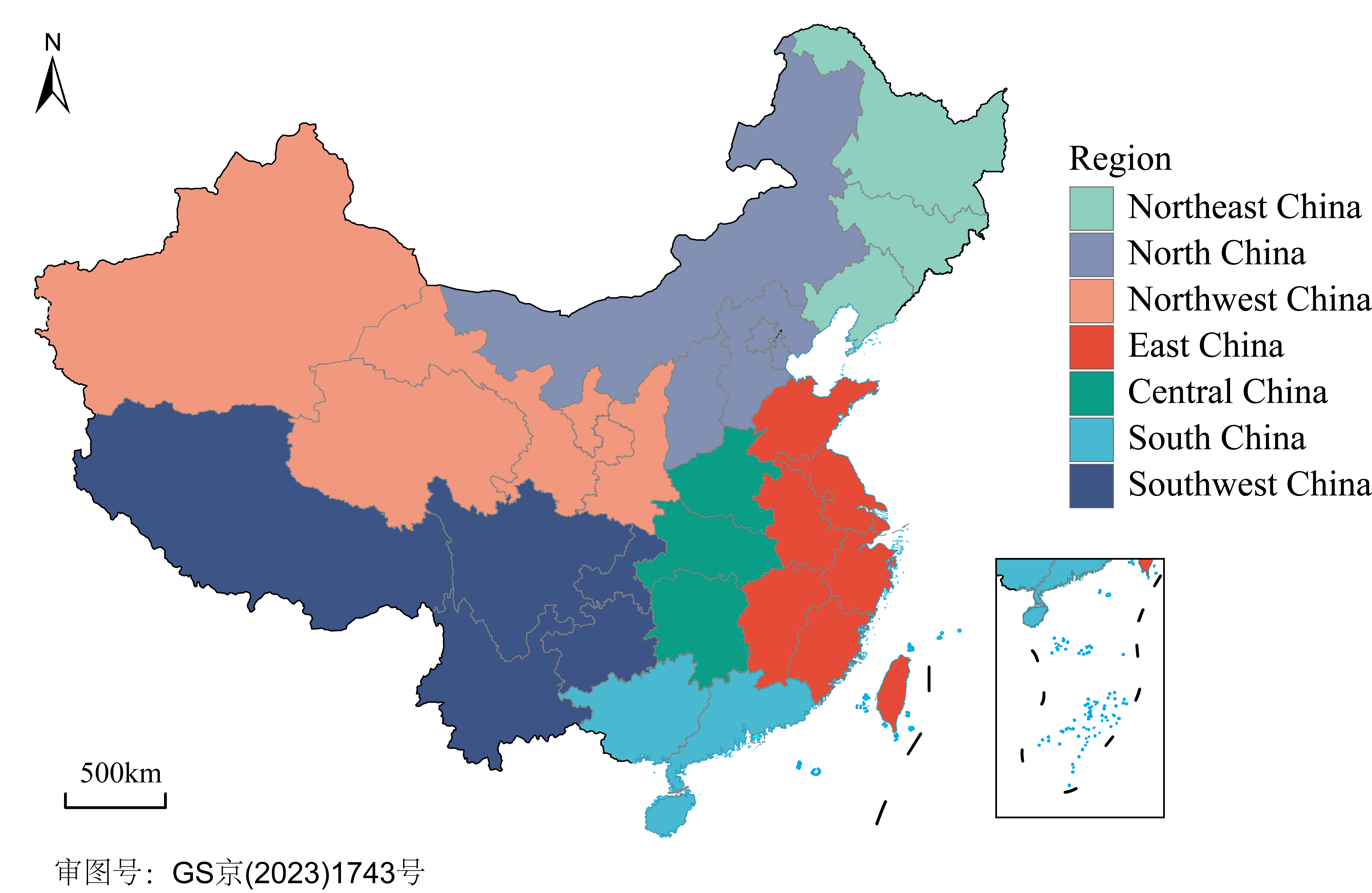


**Fig. S1.** The geographical administrative division in China.

**2.2 Estimating the number of indoor workers protected by air conditioning**

We used China’s AC (air conditioning) penetration rate to estimate the fraction of indoor workers with access to AC. Specifically, we excluded those indoor workers (including manufacturing and service workers) protected by AC systems and obtained the exposed population [17]. Here is the calculation formula.

$${Pop\_adjusted}_{ij}= {pop}_{ij}\times(1-{Penetration\_rate}_{i})$$

${pop}_{ij}$ denotes the total number of indoor workers in year $i$ and grid $j$*.* ${Penetration\_rate}_{i}$ denotes the average penetration rate of AC systems in China. ${Pop\_adjusted}_{ij}$ denotes the actual number of indoor workers who are not protected by AC systems. Since the AC penetration rate in the workplace was not available, we used the household AC penetration rate instead, which was a common method used in previous studies [17].

The average AC penetration rate in the historical periods was from the data on the ownership of AC systems per 100 households from the China Statistical Yearbook. The average AC penetration rate has reached almost 60%, which is consistent with the rate reported by the International Energy Agency (<https://www.iea.org/data-and-statistics/charts/percentage-of-households-equiped-with-ac-in-selected-countries-2018>). We assumed future AC penetration rate is constant (60%), and estimated associated WHL in the text.

Considering that the future AC penetration rate may increase with China's rapid economic development, we added the uncertainty analysis of different future AC penetration rates. Previous projection studies suggested that the average AC penetration rate in China will be almost saturated by the end of the century, depending on the pace of economic growth [18, 19]. Therefore, we considered three scenarios of future AC penetration rates, increasing by 10%, 20%, and 30% to reach 70%, 80%, and 90% by the end of the century.

**2.3 Reason for aggregating gridded data to 50*50 km for analysis**

We derived climate data from the regional climate model at a 25*25km resolution, population data under different fertility rates at a 1$\times$1 km resolution, and future GDP data at a 50*50km resolution.

To obtain the gridded working population, we need to multiply the gridded total population by the proportion of the population living in urban and rural areas, and then multiply by the employment rate in each sector in urban or rural areas. However, employment rate data of 1km*1km (or 25*25km) is not available, as Chinese statistical yearbooks only provide employment rates at the provincial level. Therefore, we had to reduce the spatial resolution for further analysis. To be more policy-relevant, we aggregated the gridded WHL to the provincial level for comparison, rather than pursuing a very high spatial resolution. 50km*50km resolution can meet the needs for provincial analysis. In addition, 50*50km resolution can also match the GDP data.

1. **Development of ERFs between WBGT and labor productivity**

**3.1 The global widely-used ERF**

The global widely-used ERF (short for “global function”) developed by Tord Kjellstrom et al. (2018) describes the relationship between WBGT and productivity loss for different levels of work intensity (Fig. S2) [10]. The formula is as follows.

$Loss fraction=\frac{1}{2}\times\left( 1+erf\left( \frac{WBGT-\mathrm{Prod}_{\mathrm{mean}}}{\mathrm{Prod}_{\mathrm{sd}}} \right) \right)$ (1.1)

The $erf$ is a cumulative distribution function. $\mathrm{Prod}_{\mathrm{mean}}$ and $\mathrm{Prod}_{\mathrm{sd}}$ are function parameters for working with different intensities of 200W, 300W and 400W (Table S1). The $Loss fraction$ is the percentage of work time lost to the total work time.


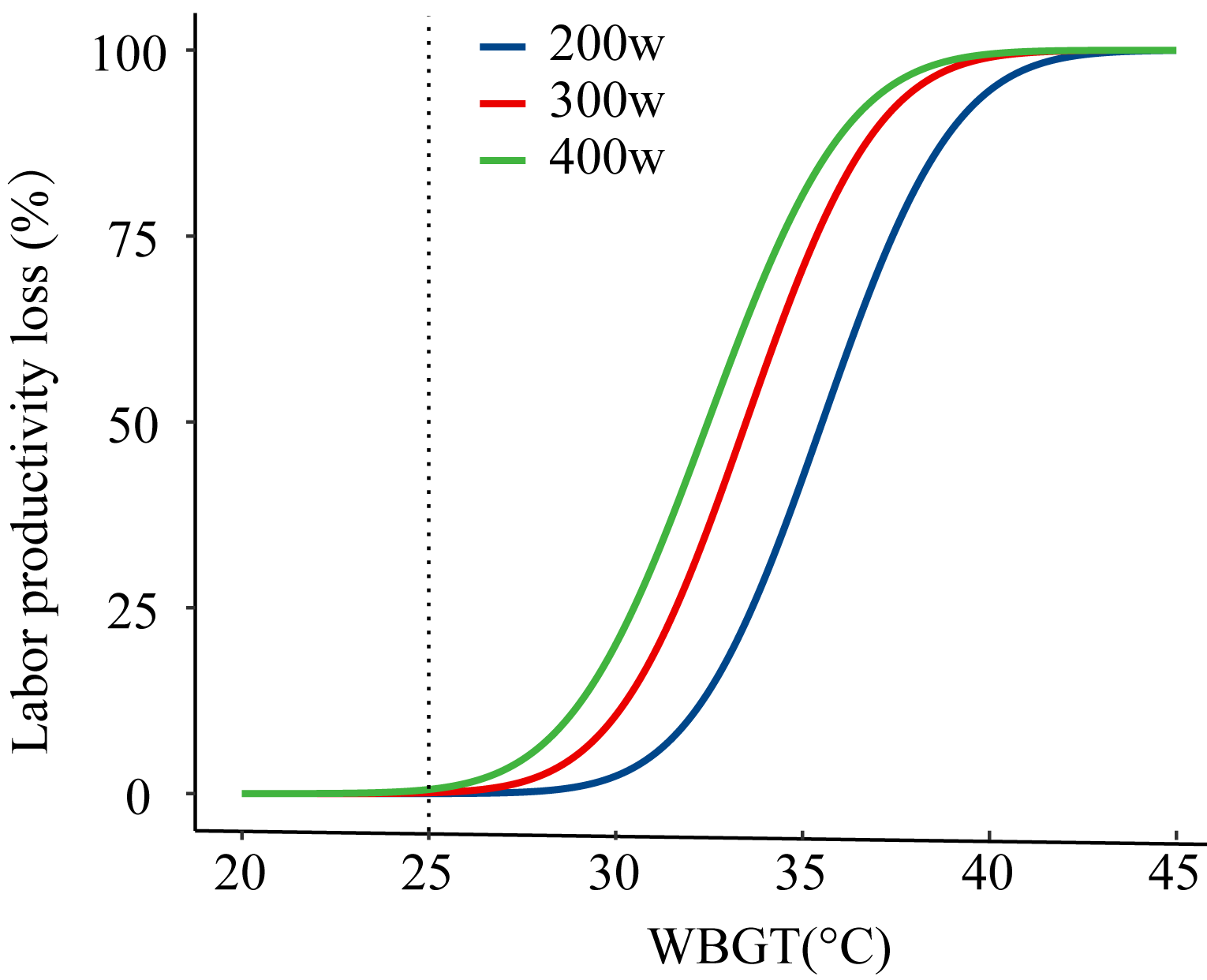


**Fig. S2.** The exposure-response curves between WBGT and productivity loss are based on the ERF developed by Tord Kjellstrom. The 200, 300 and 400w represent low, moderate and high work intensity, respectively. 25 °C is identified as the WBGT threshold for labor loss in the study.

**Table S1.** Function parameters for the ERF between WBGT and productivity loss developed by Tord Kjellstrom.

| Work intensity level | $\mathrm{Prod}_{\mathrm{mean}}$ | $\mathrm{Prod}_{\mathrm{sd}}$ |
| --- | --- | --- |
| 200W | 35.533 | 3.948 |
| 300W | 33.492 | 3.948 |
| 400W | 32.465 | 4.161 |

W is short for Watts. The 200, 300, and 400 Watts represent low, moderate, and high work intensity respectively.

**3.2 The ERF adjusted by the Chinese occupational health standard**

Previous studies showed that heat sensitivity varied among workers from different regions [20]. Occupational health standards are different in different countries, and they were developed by considering the heat sensitivity of local workers. We adjusted the global ERF using localized Chinese occupational health standards, which better reflect the heat sensitivity of the Chinese population. The occupational health standard was issued by the Chinese government with reliable and credible data sources.

We first fitted exposure-response curves for different labor intensities (200W, 300W, and 400W) by using the distribution cumulative function, based on the recommended work/rest ratios in the Chinese occupational health standard (Fig.3A). The recommended work/rest ratios can be found at the “Occupational exposure limits for hazardous agents in the workplace, Part2: Physical agents (GBZ 2.2-2007)”, which was issued by National Health Commission in 2007 [11]. The statistical formula of the ERF developed by occupational health standard is the same as formula 1.1, but with different function parameters (Table S2). We also calculated the differences in function parameters ($\mathrm{Prod}_{\mathrm{mean}}$ and $\mathrm{Prod}_{\mathrm{sd}}$) of 200W and 400W compared with the 300W, respectively.

$Loss fraction=\frac{1}{2}\times\left( 1+erf\left( \frac{WBGT-\mathrm{Prod}_{\mathrm{mean}}}{\mathrm{Prod}_{\mathrm{sd}}} \right) \right)$ (1.2)

Secondly, compared with the ERF developed by the occupational health standard, the ERF developed by epidemiological surveys would give a more reliable estimate. As the exposure-response curve of 300W in the global ERF was developed by epidemiological surveys, we used the curve of 300W and the corresponding function parameters in our Chinese epidemiological ERF. To further estimate the exposure-response curves and function parameters of 200W and 400W, we used the differences in parameters between the three curves in the first step to extrapolate the epidemiological exposure-response curves of 200W and 400W for Chinese epidemiological ERF. The function parameters for the Chinese ERF adjusted by the occupational health standard were in Table S3, and the exposure-response curves were in Figure S3B. We found that the Chinese exposure-response curves are slightly lower than the global curves under the common WBGT range (20°C ≤ WBGT ≤ 34°C).

**Table S2.** Function parameters for the ERF based on the Chinese occupational health standard.

| Work intensity level | $\mathrm{Prod}_{\mathrm{mean}}$ | $\mathrm{Prod}_{\mathrm{sd}}$ |
| --- | --- | --- |
| 200W | 31.987 | 1.612 |
| 300W | 30.350 | 2.057 |
| 400W | 29.350 | 2.057 |

W is short for Watts. The 200, 300, and 400 Watts represent low, moderate, and high work intensity respectively.

**Table S3**. Function parameters for the Chinese ERF adjusted by the occupational health standard.

| Work intensity level | $\mathrm{Prod}_{\mathrm{mean}}$ | $\mathrm{Prod}_{\mathrm{sd}}$ |
| --- | --- | --- |
| 200W | 35.129 | 3.503 |
| 300W | 33.492 | 3.948 |
| 400W | 32.492 | 3.948 |

W is short for Watts. The 200, 300, and 400 Watts represent low, moderate, and high work intensity respectively.


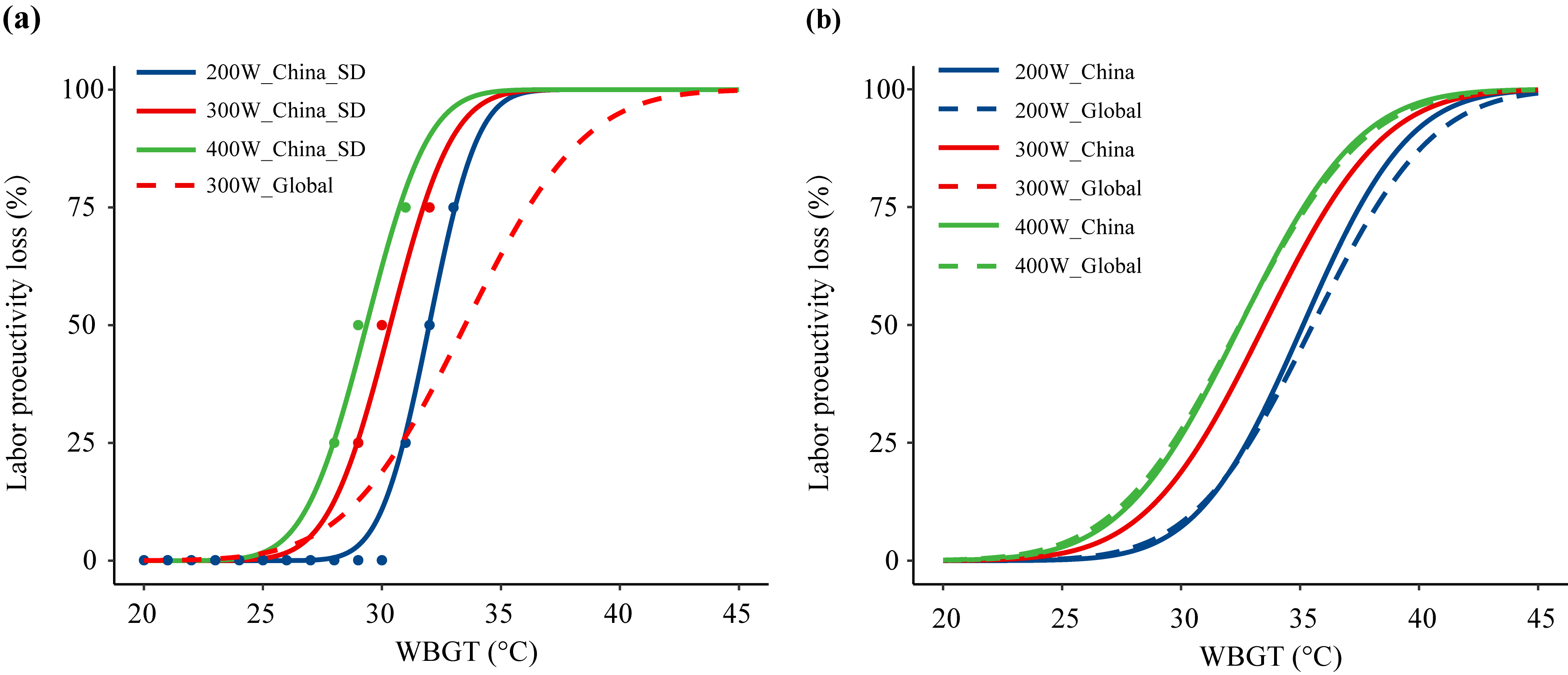


**Fig. S3.** a. The exposure-response curves between WBGT and productivity loss based on the Chinese occupational health standards. b. The exposure-response curves between WBGT and productivity loss based on the adjusted ERF and global ERF. China_SD represents the curve developed by the Chinese occupational health standard. The dots in the left panel represent the recommended work/rest ratios by Chinese occupational health standards.

1. **Projecting future labor losses due to heat stress**

**4.1 Estimating drivers for changes in future WHL**

To understand WHL changes in different climate change scenarios, we referred to the factor separation method to estimate the main drivers [21]. We considered three important drivers including the climate effects, population effects, and their interaction effects. In other words, the total effect is the sum of the three components. Here is a detailed explanation of the three components:

The climate effect is the impact on labor productivity when WBGT rises from the 2020s to 2100s levels, while controlling for other variables and holding constant the working population. It revealed the contribution of climate factors to the results. The population effect is the impact on labor productivity when the working population changed from the 2020s to 2100s levels, while controlling for other variables and holding climate constant. It revealed the contribution of population factors to the results. The interaction effect is the remaining effect after subtracting the climate and population effects from the total effect. It indicates the impact on labor productivity when WBGT and population are simultaneously changed from the 2020s to 2100s levels. For example, if the interaction effect is positive, it suggests that some regions will face an increase in population in parallel with an increase in WBGT, leading to an increase in labor productivity losses.

Specifically, we isolated the impacts of population and climate by recalculating WHL when one factor was held constant. The climate effect is the direct effect of changing temperature from 2020 to 2100 levels with other variables held constant. The climate effect is the direct effect of changing population from 2020 to 2100 levels with other variables held constant. The interaction effect occurs when temperature and population simultaneously changed from 2020 to 2100 levels. The formula is as follows:

$$Population effects={SIM}_{population}-{SIM}_{control}$$

$$Climate effects= {SIM}_{climate}-{SIM}_{control}$$

$$Interaction effects = {SIM}_{population+climate}-{SIM}_{population}-{SIM}_{climate}+{SIM}_{control}$$

The definition of ${SIM}_{population}$, ${SIM}_{population}$, and ${SIM}_{control}$ are in Table S4. ${SIM}_{control}$ is the WHL in the baseline period with climate forcing in the 2020s and working population in the 2020s.${SIM}_{climate}$ is the WHL based on climate forcing in the 2090s and working population in the 2020s; ${SIM}_{population}$ is the WHL based on climate forcing in the 2020s and working population in the 2090s; ${SIM}_{population+climate}$ is the WHL based on climate forcing in the 2090s and working population in the 2090s. We estimated the gridded WHL in different simulations, and calculated the climate effects, population effects and their interaction effects on future WHL based on the above formulas.

**Table S4.** Description of the simulations for exploring drivers for changes in future WHL.

| Simulations | Simulation Description |
| --- | --- |
| ${SIM}_{control}$ | Climate in the 2020s and working population in the 2020s |
| ${SIM}_{climate}$ | Climate in the 2090s and working population in the 2020s |
| ${SIM}_{population}$ | Climate in the 2020s and working population in the 2090s |
| ${SIM}_{population+climate}$ | Climate in the 2090s and working population in the 2090s |

**4.2 Estimating economic costs of avoided WHL**

In the study, we used the human capital (HC) method to estimate the economic cost of avoided WHL [22, 23]. Specifically, the economic cost was the product of the avoided WHL and its unit value. The calculation formula is as follows.

${Economic\_costs}_{ij}= {Avoided\_WHL}_{ij}\times{GDP\_per\_hour}_{ij}$

Here ${Avoided\_WHL}_{ij}$ denotes the annual WHL in year $i$ and province $j$*.* ${GDP\_per\_hour}_{ij}$ represents the GDP per work hour in year $i$ and province $j$. ${GDP\_per\_hour}_{ij}$ is equal to the total GDP divided by the total number of possible work hours in a year (possible work hours in a year = number of the working population * 365 days * 8 h/day).

To estimate the economic cost, we need to collect historical and future GDP in each province per year. Historical GDP was from China Statistical Yearbooks. The future GDP in each province from 2021 to 2100 was from the gridded datasets for population and economy under Shared Socioeconomic Pathways in China [14]. The datasets were developed using the Population-Development-Environment (PDE) model and the Cobb-Douglas production model, incorporating both localized population and economic parameters. Future GDP under SSP2 was used, since SSP2 was also the scenario for projecting the future population in the study.

1. **Uncertainty analysis**

Workers usually work overtime to get more pay, especially in large and densely populated cities. In China, workers usually don’t go to work early, but postpone the time of getting off work. Therefore, we have added the uncertainty analysis of different delayed working times, considering two common scenarios including a 1-hour delay and a 2-hour delay. In the previous analysis, we assumed a worker works 8 hours a day (2 hours at WBGTmean, 2 hours at WBGTmax, and 4 hours at WBGThalf). Considering that the WBGT in the early evening is close to WBGTmean [10], we assumed a worker works 9 hours a day (3 hours at WBGTmean, 2 hours at WBGTmax, and 4 hours at WBGThalf), and works 10 hours a day (4 hours at WBGTmean, 2 hours at WBGTmax, and 4 hours at WBGThalf).

**Supplementary Results**

**1. Temporal trends in four** **meteorological indicators**

Compared to the baseline period (1986-2005), ambient temperature and relative humidity will increase, and wind speed and radiation will decrease in the future. For example, the annual temperature will show an increase of 4.4°C (4.3-4.5°C) by the end of the century under RCP8.5, which is 4.3 times higher than under RCP2.6 (Fig. S4).


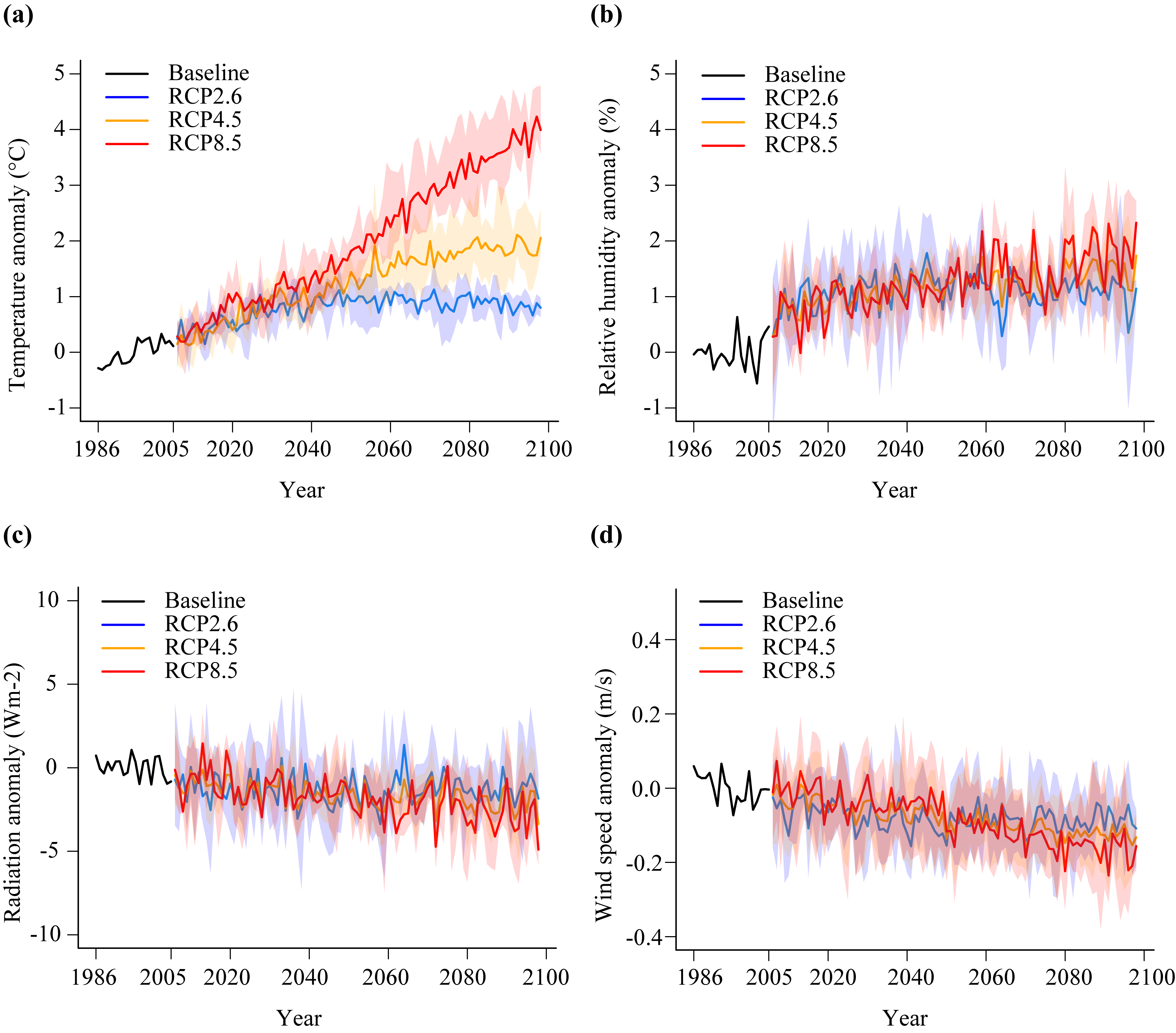


**Fig. S4.** Changes in the annual ambient temperature, relative humidity, radiation, and wind speed under different RCP scenarios. Baseline period: 1986-2005. RCP2.6, RCP4.5 and RCP8.5 denote the low, medium and high emission scenarios, respectively. The shaded areas represent the range for different GCMs.

**2. Change in future working population under different fertility scenarios**

The total population in China will peak in the 2020s and then decline in the second half of this century (Fig S5-a). The proportion of the population living in urban areas will gradually increase (63.4% in the 2020s and 80.0% in the 2090s), while the proportion of the population living in rural areas will gradually decrease (Figure S5b). On this basis, future employment rates will be dynamic. For example, the share of the population employed in agriculture will decrease from 24.5% in the 2020s to 12.4% in the 2090s under the medium fertility scenario (Figure S5-d). Overall, the working population will peak in the 2020s, with an annual average of 0.75 billion workers under the three fertility scenarios. By the end of the century, the annual working population will decline to 0.42, 0.49, and 0.55 billion under the low, medium, and high fertility scenarios, respectively (Fig S5-cde).

**
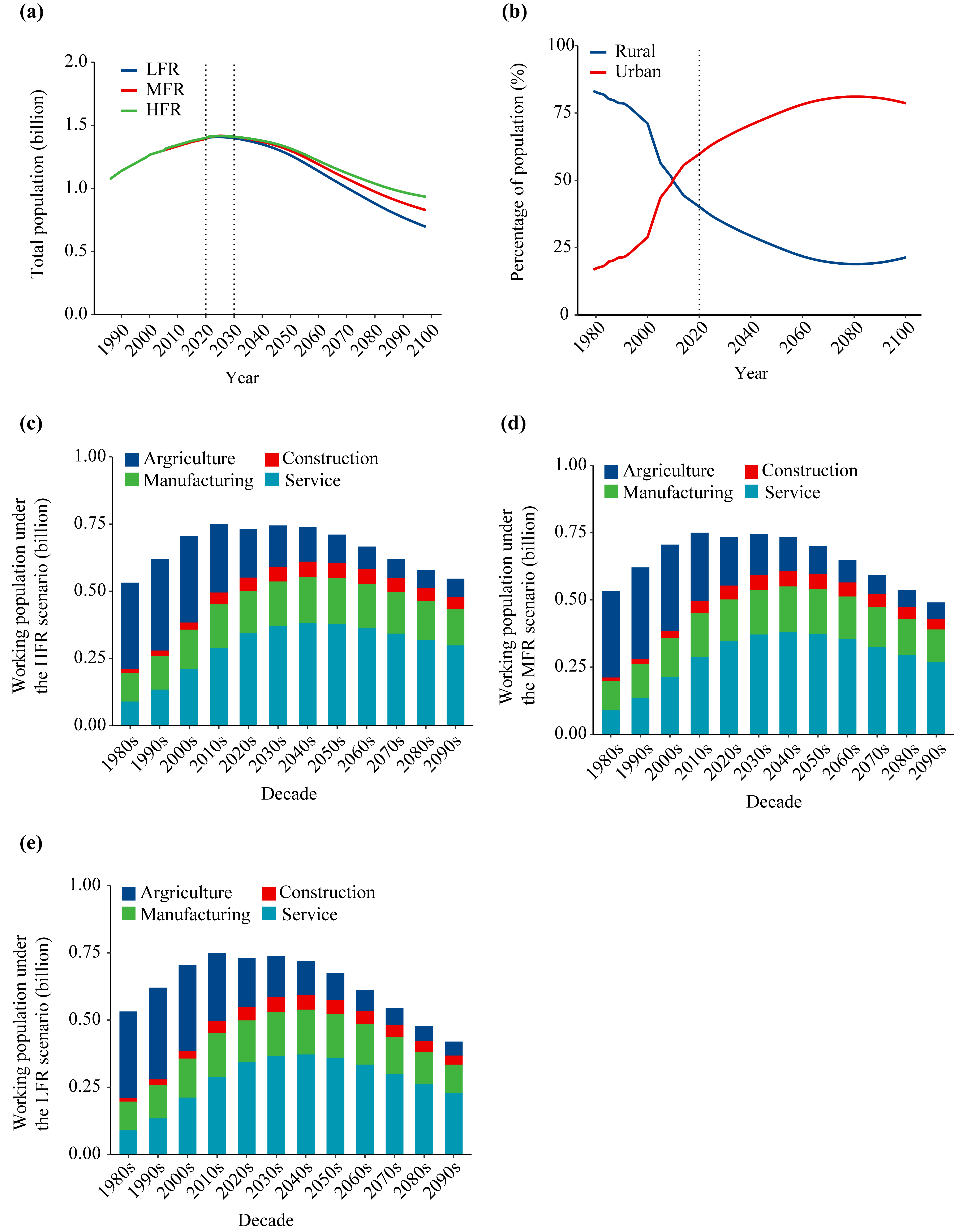
**

**Fig. S5.** Changes in the total population and the working population under scenarios with different fertility rates. a, The total population under different fertility rates scenarios. b, Percentages of the population living in urban or rural areas in the future. c-e, The working population by sector under the high, middle, and low fertility rate scenarios. HFR: high fertility rate. MFR: middle fertility rate. LFR: low fertility rate.

**3. Drivers of changes in WHL in early, middle and late future periods**

In the early future, there will be net increases in WHL under RCP4.5 and RCP8.5. In the middle and late future periods, there will be net decreases in WHL under RCP2.6 and RCP4.5, whereas there will be net increases in WHL under RCP8.5. The declining population is not sufficient to offset the detrimental effects of rising temperature under the RCP8.5 scenario (Table S5).

**Table S5.** Drivers of future changes in WHL under different climate change scenarios.

| Drivers | RCP2.6 | RCP4.5 | RCP8.5 |
| --- | --- | --- | --- |
| ***Full future period*** | | | |
| Net change | -10.58 | 1.97 | 21.75 |
| Climate effects | 0.27 | 18.59 | 53.30 |
| Population effects | -10.77 | -9.90 | -10.62 |
| Interaction effects | -0.08 | -6.72 | -20.93 |
| ***Early future period*** | | | |
| Net change | -0.22 | 2.29 | 1.72 |
| Climate effects | 0.70 | 3.22 | 2.70 |
| Population effects | -0.91 | -0.84 | -0.90 |
| Interaction effects | -0.01 | -0.09 | -0.08 |
| ***Middle future period*** | | | |
| Net change | -1.65 | -1.24 | 4.07 |
| Climate effects | 0.72 | 1.63 | 8.01 |
| Population effects | -2.32 | -2.72 | -3.21 |
| Interaction effects | -0.05 | -0.15 | -0.73 |
| ***Late future period*** | | | |
| Net change | -2.46 | -0.01 | 3.03 |
| Climate effects | -1.42 | 1.70 | 6.56 |
| Population effects | -1.15 | -1.58 | -3.04 |
| Interaction effects | 0.11 | -0.13 | -0.49 |

Unit: Billion hours. Early future:2021-2040. Middle future:2051-2070. Late future:2081-2100.

**4. Future WHL by the province in early, middle and late future periods**

The provinces in the southern, eastern and central regions will have the largest WHL in the early, middle, and late future periods in China (Table S6-S8). Guangdong is at the top of the list with 3.5 billion hours, 4.8 billion hours and 5.9 billion hours of annual WHL in the early, middle, and late future periods under the RCP8.5 scenario, respectively.

**Table S6.** Future WHL by province in the early future in China.

| **Province** | **RCP2.6** | | **RCP4.5** | | **RCP8.5** | |
| --- | --- | --- | --- | --- | --- | --- |
|  | **Rank** | **WHL (%)** | **Rank** | **WHL (%)** | **Rank** | **WHL (%)** |
| Guangdong | 1 | 3.4 (13.2%) | 1 | 3.2 (12.7%) | 1 | 3.5 (13.4%) |
| Henan | 2 | 2.3 (8.9%) | 2 | 2.2 (8.9%) | 2 | 2.5 (9.5%) |
| Guangxi | 3 | 2.3 (8.8%) | 3 | 2.2 (8.8%) | 3 | 2.3 (8.6%) |
| Hunan | 4 | 2.1 (8.1%) | 4 | 2.1 (8.2%) | 4 | 2.1 (8.0%) |
| Hubei | 5 | 2.1 (8.1%) | 5 | 2.0 (8.0%) | 5 | 2.0 (7.7%) |
| Sichuan | 6 | 2.1 (8.0%) | 6 | 2.0 (8.0%) | 6 | 2.1 (7.8%) |
| Jiangsu | 7 | 2.1 (8.0%) | 7 | 2.0 (7.9%) | 7 | 2.0 (7.6%) |
| Anhui | 8 | 1.6 (6.2%) | 8 | 1.5 (6.1%) | 8 | 1.5 (5.9%) |
| Jiangxi | 9 | 1.4 (5.5%) | 9 | 1.4 (5.5%) | 9 | 1.4 (5.3%) |
| Shandong | 10 | 1.4 (5.3%) | 10 | 1.4 (5.4%) | 10 | 1.4 (5.4%) |
| Chongqing | 11 | 1.1 (4.2%) | 11 | 1.0 (4.2%) | 11 | 1.1 (4.1%) |
| Zhejiang | 12 | 0.9 (3.5%) | 12 | 0.9 (3.5%) | 12 | 0.9 (3.5%) |
| Hebei | 13 | 0.8 (3.3%) | 13 | 0.8 (3.4%) | 13 | 0.9 (3.4%) |
| Fujian | 14 | 0.5 (2.0%) | 14 | 0.5 (2.0%) | 14 | 0.6 (2.1%) |
| Shaanxi | 15 | 0.3 (1.2%) | 15 | 0.3 (1.2%) | 15 | 0.3 (1.2%) |
| Guizhou | 16 | 0.3 (1.0%) | 16 | 0.3 (1.0%) | 16 | 0.3 (1.1%) |
| Hainan | 17 | 0.2 (0.9%) | 17 | 0.2 (0.9%) | 17 | 0.3 (1.0%) |
| Shanghai | 18 | 0.2 (0.8%) | 18 | 0.2 (0.8%) | 18 | 0.2 (0.8%) |
| Tianjin | 19 | 0.1 (0.5%) | 20 | 0.1 (0.5%) | 20 | 0.1 (0.5%) |
| Beijing | 20 | 0.1 (0.5%) | 19 | 0.1 (0.5%) | 19 | 0.1 (0.5%) |
| Liaoning | 21 | 0.1 (0.5%) | 21 | 0.1 (0.5%) | 21 | 0.1 (0.5%) |
| Shanxi | 22 | 0.1 (0.4%) | 22 | 0.1 (0.4%) | 22 | 0.1 (0.4%) |
| Yunnan | 23 | 0.1 (0.3%) | 24 | 0.1 (0.3%) | 24 | 0.1 (0.4%) |
| Heilongjiang | 24 | 0.1 (0.3%) | 23 | 0.1 (0.3%) | 23 | 0.1 (0.3%) |
| Jilin | 25 | 0.1 (0.2%) | 25 | 0.1 (0.2%) | 25 | 0.1 (0.2%) |
| Taiwan | 26 | 0.0 (0.2%) | 26 | 0.0 (0.2%) | 26 | 0.1 (0.2%) |
| Nei Mongol | 27 | 0.0 (0.2%) | 27 | 0.0 (0.2%) | 27 | 0.0 (0.2%) |
| Gansu | 28 | 0.0 (0.1%) | 29 | 0.0 (0.1%) | 29 | 0.0 (0.1%) |
| Hongkong | 29 | 0.0 (0.0%) | 28 | 0.0 (0.1%) | 28 | 0.0 (0.1%) |
| Xinjiang | 30 | 0.0 (0.0%) | 30 | 0.0 (0.0%) | 30 | 0.0 (0.0%) |
| Ningxia | 31 | 0.0 (0.0%) | 31 | 0.0 (0.0%) | 31 | 0.0 (0.0%) |
| Macau | 32 | 0.0 (0.0%) | 32 | 0.0 (0.0%) | 32 | 0.0 (0.0%) |
| Tibet | 33 | 0.0 (0.0%) | 33 | 0.0 (0.0%) | 33 | 0.0 (0.0%) |
| Qinghai | 34 | 0.0 (0.0%) | 34 | 0.0 (0.0%) | 34 | 0.0 (0.0%) |

RCP: Representative Concentration Pathways. The unit of WHL is billion hours. The proportion represents the percentage of WHL to total WHL in all 31 provinces. The 0.00 in and out of brackets represents the value is less than 0.001, but not equal to 0.

**Table S7.** Future WHL by province in the middle future in China.

| **Province** | **RCP2.6** | | **RCP4.5** | | **RCP8.5** | |
| --- | --- | --- | --- | --- | --- | --- |
|  | **Rank** | **WHL (%)** | **Rank** | **WHL (%)** | **Rank** | **WHL (%)** |
| Guangdong | 1 | 2.9 (13.4%) | 1 | 3.5 (13.4%) | 1 | 4.8 (13.8%) |
| Henan | 2 | 2.2 (10.0%) | 2 | 2.6 (10.0%) | 2 | 3.5 (10.1%) |
| Guangxi | 3 | 2.0 (9.0%) | 3 | 2.3 (8.7%) | 3 | 3.0 (8.7%) |
| Hunan | 4 | 1.9 (8.7%) | 4 | 2.3 (8.5%) | 4 | 2.9 (8.2%) |
| Jiangsu | 5 | 1.7 (7.7%) | 5 | 2.0 (7.6%) | 5 | 2.6 (7.4%) |
| Hubei | 6 | 1.6 (7.4%) | 6 | 1.9 (7.1%) | 7 | 2.4 (6.8%) |
| Sichuan | 7 | 1.5 (7.0%) | 7 | 1.8 (6.8%) | 6 | 2.4 (6.8%) |
| Anhui | 8 | 1.4 (6.3%) | 8 | 1.6 (6.1%) | 8 | 2.1 (5.9%) |
| Jiangxi | 9 | 1.3 (5.7%) | 9 | 1.5 (5.7%) | 9 | 1.9 (5.5%) |
| Shandong | 10 | 1.1 (4.9%) | 10 | 1.3 (5.0%) | 10 | 1.8 (5.0%) |
| Chongqing | 11 | 0.9 (4.2%) | 11 | 1.1 (4.2%) | 11 | 1.4 (4.0%) |
| Zhejiang | 12 | 0.8 (3.6%) | 12 | 1.0 (3.7%) | 12 | 1.3 (3.7%) |
| Hebei | 13 | 0.8 (3.5%) | 13 | 0.9 (3.5%) | 13 | 1.3 (3.6%) |
| Fujian | 14 | 0.4 (2.0%) | 14 | 0.6 (2.2%) | 14 | 0.8 (2.3%) |
| Guizhou | 15 | 0.3 (1.2%) | 15 | 0.4 (1.4%) | 15 | 0.6 (1.7%) |
| Shaanxi | 16 | 0.2 (1.0%) | 16 | 0.3 (1.0%) | 16 | 0.4 (1.1%) |
| Hainan | 17 | 0.2 (0.9%) | 17 | 0.3 (1.0%) | 17 | 0.4 (1.0%) |
| Shanghai | 18 | 0.2 (0.7%) | 18 | 0.2 (0.8%) | 18 | 0.3 (0.8%) |
| Tianjin | 19 | 0.1 (0.5%) | 20 | 0.1 (0.6%) | 20 | 0.2 (0.6%) |
| Beijing | 20 | 0.1 (0.5%) | 19 | 0.2 (0.6%) | 19 | 0.2 (0.6%) |
| Liaoning | 21 | 0.1 (0.3%) | 21 | 0.1 (0.4%) | 22 | 0.2 (0.4%) |
| Shanxi | 22 | 0.1 (0.3%) | 22 | 0.1 (0.3%) | 23 | 0.1 (0.4%) |
| Yunnan | 23 | 0.1 (0.3%) | 23 | 0.1 (0.3%) | 21 | 0.2 (0.5%) |
| Heilongjiang | 24 | 0.0 (0.2%) | 25 | 0.1 (0.2%) | 25 | 0.1 (0.2%) |
| Taiwan | 25 | 0.0 (0.2%) | 24 | 0.1 (0.2%) | 24 | 0.1 (0.2%) |
| Jilin | 26 | 0.0 (0.2%) | 26 | 0.0 (0.2%) | 26 | 0.1 (0.2%) |
| Nei Mongol | 27 | 0.0 (0.1%) | 27 | 0.0 (0.1%) | 27 | 0.0 (0.1%) |
| Hongkong | 28 | 0.0 (0.1%) | 28 | 0.0 (0.1%) | 28 | 0.0 (0.1%) |
| Gansu | 29 | 0.0 (0.0%) | 30 | 0.0 (0.1%) | 30 | 0.0 (0.1%) |
| Ningxia | 30 | 0.0 (0.0%) | 31 | 0.0 (0.0%) | 31 | 0.0 (0.1%) |
| Xinjiang | 31 | 0.0 (0.0%) | 29 | 0.0 (0.1%) | 29 | 0.0 (0.1%) |
| Macau | 32 | 0.0 (0.0%) | 32 | 0.0 (0.0%) | 32 | 0.0 (0.0%) |
| Tibet | 33 | 0.0 (0.0%) | 33 | 0.0 (0.0%) | 33 | 0.0 (0.0%) |
| Qinghai | 34 | 0.0 (0.0%) | 34 | 0.0 (0.0%) | 34 | 0.0 (0.0%) |

RCP: Representative Concentration Pathways. The unit of WHL is billion hours. The proportion represents the percentage of WHL to total WHL in all 31 provinces. The 0.00 in and out of brackets represents the value is less than 0.001, but not equal to 0.

**Table S8**. Future WHL by province in the late future in China.

| **Province** | **RCP2.6** | | **RCP4.5** | | **RCP8.5** | |
| --- | --- | --- | --- | --- | --- | --- |
|  | **Rank** | **WHL (%)** | **Rank** | **WHL (%)** | **Rank** | **WHL (%)** |
| Guangdong | 1 | 2.0 (12.6%) | 1 | 2.9 (12.2%) | 1 | 5.9 (12.9%) |
| Henan | 2 | 2.0 (12.6%) | 2 | 2.8 (12.0%) | 2 | 5.1 (11.0%) |
| Guangxi | 3 | 1.5 (9.3%) | 3 | 2.2 (9.5%) | 3 | 4.3 (9.3%) |
| Hunan | 4 | 1.5 (9.1%) | 4 | 2.0 (8.6%) | 4 | 3.7 (8.0%) |
| Hubei | 5 | 1.2 (7.2%) | 5 | 1.6 (6.8%) | 5 | 2.9 (6.4%) |
| Jiangsu | 6 | 1.1 (6.9%) | 6 | 1.6 (6.8%) | 6 | 3.2 (6.9%) |
| Anhui | 7 | 1.1 (6.5%) | 7 | 1.5 (6.3%) | 7 | 2.8 (6.2%) |
| Jiangxi | 8 | 1.0 (6.0%) | 9 | 1.3 (5.7%) | 9 | 2.5 (5.5%) |
| Sichuan | 9 | 0.9 (5.8%) | 8 | 1.4 (5.9%) | 8 | 2.6 (5.7%) |
| Chongqing | 10 | 0.8 (4.7%) | 10 | 1.1 (4.7%) | 10 | 2.0 (4.4%) |
| Shandong | 11 | 0.7 (4.1%) | 11 | 1.1 (4.7%) | 11 | 2.2 (4.9%) |
| Hebei | 12 | 0.6 (3.6%) | 12 | 0.9 (4.0%) | 12 | 1.7 (3.8%) |
| Zhejiang | 13 | 0.5 (3.2%) | 13 | 0.8 (3.2%) | 13 | 1.6 (3.4%) |
| Guizhou | 14 | 0.3 (1.9%) | 14 | 0.6 (2.3%) | 14 | 1.3 (2.9%) |
| Fujian | 15 | 0.3 (1.9%) | 15 | 0.5 (2.0%) | 15 | 1.0 (2.3%) |
| Hainan | 16 | 0.2 (1.2%) | 16 | 0.3 (1.2%) | 16 | 0.5 (1.2%) |
| Shaanxi | 17 | 0.1 (0.8%) | 17 | 0.2 (0.9%) | 17 | 0.5 (1.0%) |
| Shanghai | 18 | 0.1 (0.5%) | 18 | 0.1 (0.6%) | 18 | 0.3 (0.7%) |
| Tianjin | 19 | 0.1 (0.5%) | 19 | 0.1 (0.5%) | 19 | 0.2 (0.5%) |
| Beijing | 20 | 0.1 (0.4%) | 20 | 0.1 (0.5%) | 20 | 0.2 (0.5%) |
| Yunnan | 21 | 0.0 (0.3%) | 21 | 0.1 (0.4%) | 21 | 0.3 (0.7%) |
| Shanxi | 22 | 0.0 (0.2%) | 22 | 0.1 (0.3%) | 22 | 0.2 (0.4%) |
| Liaoning | 23 | 0.0 (0.1%) | 23 | 0.1 (0.2%) | 23 | 0.1 (0.3%) |
| Taiwan | 24 | 0.0 (0.1%) | 24 | 0.0 (0.2%) | 24 | 0.1 (0.2%) |
| Hongkong | 25 | 0.0 (0.1%) | 25 | 0.0 (0.1%) | 25 | 0.1 (0.2%) |
| Jilin | 26 | 0.0 (0.1%) | 26 | 0.0 (0.1%) | 26 | 0.1 (0.1%) |
| Heilongjiang | 27 | 0.0 (0.1%) | 28 | 0.0 (0.1%) | 28 | 0.1 (0.1%) |
| Nei Mongol | 28 | 0.0 (0.1%) | 29 | 0.0 (0.1%) | 29 | 0.1 (0.1%) |
| Xinjiang | 29 | 0.0 (0.1%) | 27 | 0.0 (0.1%) | 27 | 0.1 (0.2%) |
| Ningxia | 30 | 0.0 (0.0%) | 30 | 0.0 (0.1%) | 30 | 0.0 (0.1%) |
| Gansu | 31 | 0.0 (0.0%) | 31 | 0.0 (0.1%) | 31 | 0.0 (0.1%) |
| Macau | 32 | 0.0 (0.0%) | 32 | 0.0 (0.0%) | 32 | 0.0 (0.0%) |
| Tibet | 33 | 0.0 (0.0%) | 33 | 0.0 (0.0%) | 33 | 0.0 (0.0%) |
| Qinghai | 34 | 0.0 (0.0%) | 34 | 0.0 (0.0%) | 34 | 0.0 (0.0%) |

RCP: Representative Concentration Pathways. The unit of WHL is billion hours. The proportion represents the percentage of WHL to total WHL in all 31 provinces. The 0.0 in and out of brackets represents the value is less than 0.001, but not equal to 0.

**5. The WHL for outdoor and indoor workers in each province**

Table S9 shows the Future WHL for outdoor and indoor workers under the three RCP scenarios by provinces in China. The WHL of outdoor workers will be much higher than that of indoor workers in most provinces.

**Table S9.** Future WHL for outdoor and indoor workers under the three RCP scenarios by provinces in China.

| Province | RCP2.6 | | RCP4.5 | | RCP8.5 | |
| --- | --- | --- | --- | --- | --- | --- |
|  | Indoor | Outdoor | Indoor | Outdoor | Indoor | Outdoor |
| Guangdong | 1.57 | 1.27 | 1.82 | 1.43 | 2.89 | 1.86 |
| Henan | 0.59 | 1.60 | 0.73 | 1.85 | 1.18 | 2.48 |
| Guangxi | 0.75 | 1.15 | 0.94 | 1.25 | 1.43 | 1.66 |
| Hunan | 0.62 | 1.30 | 0.76 | 1.42 | 1.13 | 1.78 |
| Hubei | 0.58 | 1.08 | 0.69 | 1.15 | 1.00 | 1.42 |
| Jiangsu | 0.53 | 1.14 | 0.64 | 1.25 | 0.97 | 1.60 |
| Anhui | 0.53 | 0.85 | 0.64 | 0.92 | 0.95 | 1.17 |
| Jiangxi | 0.51 | 0.74 | 0.62 | 0.8 | 0.93 | 1.01 |
| Sichuan | 0.42 | 1.13 | 0.52 | 1.23 | 0.79 | 1.58 |
| Shandong | 0.42 | 0.64 | 0.55 | 0.72 | 0.84 | 0.94 |
| Hebei | 0.36 | 0.39 | 0.46 | 0.43 | 0.70 | 0.58 |
| Chongqing | 0.29 | 0.65 | 0.36 | 0.72 | 0.54 | 0.93 |
| Zhejiang | 0.28 | 0.47 | 0.35 | 0.55 | 0.53 | 0.71 |
| Shanghai | 0.09 | 0.06 | 0.11 | 0.07 | 0.17 | 0.09 |
| Fujian | 0.07 | 0.35 | 0.09 | 0.44 | 0.16 | 0.64 |
| Beijing | 0.07 | 0.04 | 0.09 | 0.05 | 0.14 | 0.06 |
| Guizhou | 0.06 | 0.23 | 0.09 | 0.3 | 0.19 | 0.50 |
| Shaanxi | 0.05 | 0.17 | 0.07 | 0.19 | 0.11 | 0.28 |
| Hainan | 0.05 | 0.16 | 0.07 | 0.19 | 0.12 | 0.26 |
| Tianjin | 0.05 | 0.06 | 0.07 | 0.07 | 0.1 | 0.09 |
| Shanxi | 0.02 | 0.06 | 0.02 | 0.06 | 0.04 | 0.09 |
| Liaoning | 0.01 | 0.06 | 0.02 | 0.07 | 0.04 | 0.11 |
| Heilongjiang | 0.01 | 0.04 | 0.01 | 0.04 | 0.01 | 0.06 |
| Yunnan | 0.00 | 0.06 | 0.01 | 0.08 | 0.02 | 0.17 |
| Taiwan | 0.00 | 0.04 | 0.01 | 0.05 | 0.01 | 0.07 |
| Jilin | 0.00 | 0.03 | 0.01 | 0.04 | 0.01 | 0.06 |
| Nei Mongol | 0.00 | 0.02 | 0.01 | 0.02 | 0.01 | 0.04 |
| Gansu | 0.00 | 0.01 | 0.00 | 0.01 | 0.00 | 0.02 |
| Ningxia | 0.00 | 0.01 | 0.00 | 0.01 | 0.01 | 0.02 |
| Hongkong | 0.00 | 0.01 | 0.00 | 0.03 | 0.00 | 0.04 |
| Xinjiang | 0.00 | 0.01 | 0.00 | 0.01 | 0.01 | 0.03 |
| Macau | 0.00 | 0.00 | 0.00 | 0.00 | 0.00 | 0.01 |
| Qinghai | 0.00 | 0.00 | 0.00 | 0.00 | 0.00 | 0.00 |
| Tibet | 0.00 | 0.00 | 0.00 | 0.00 | 0.00 | 0.00 |
| Total | 7.93 | 13.83 | 9.75 | 15.45 | 15.02 | 20.36 |

RCP: Representative Concentration Pathways. The 0.00 in and out of brackets represents the value is less than 0.001, but not equal to 0.

**6. The economic costs of avoided WHL in each province**

Compared with the RCP2.6, RCP4.5, and RCP8.5 scenarios, the economic cost of annual avoided WHL was 25.7, 100.9, and 237.2 billion USD if achieving the 1.5 °C target, respectively. This is equivalent to avoiding losses of 0.1%, 0.6%, and 1.4% of annual GDP in mid-century, respectively. The avoided WHL and the associated economic cost in each province was in Table S10.

**Table S10.** The avoided WHL and associated economic costs if achieving the 1.5 °C goal in mid-century (2051-2070), compared with three RCP scenarios.

| Province | RCP2.6 | | RCP4.5 | | RCP8.5 | |
| --- | --- | --- | --- | --- | --- | --- |
|  | Avoided WHL | Economic Cost | Avoided WHL | Economic Cost | Avoided WHL | Economic Cost |
| Guangdong | 143.21 | 2.15 | 757.02 | 11.35 | 2057.56 | 30.86 |
| Henan | 184.27 | 3.19 | 535.01 | 9.27 | 1268.82 | 21.99 |
| Guangxi | 67.69 | 0.34 | 534.42 | 2.65 | 1433.95 | 7.10 |
| Jiangsu | 116.22 | 2.96 | 459.91 | 11.70 | 1047.07 | 26.63 |
| Hunan | 93.25 | 1.19 | 444.48 | 5.65 | 1057.18 | 13.45 |
| Sichuan | 115.08 | 1.21 | 400.9 | 4.21 | 986.35 | 10.35 |
| Shandong | 128.42 | 3.93 | 385.83 | 11.82 | 848.56 | 25.99 |
| Hubei | 108.99 | 1.62 | 385.53 | 5.73 | 876.34 | 13.02 |
| Anhui | 89.66 | 0.95 | 344.79 | 3.65 | 798.89 | 8.47 |
| Jiangxi | 55.83 | 0.47 | 304.11 | 2.58 | 722.80 | 6.13 |
| Hebei | 94.75 | 1.48 | 263.41 | 4.11 | 594.55 | 9.27 |
| Zhejiang | 40.91 | 1.33 | 249.53 | 8.13 | 552.67 | 18.00 |
| Chongqing | 54.62 | 0.55 | 234.07 | 2.35 | 543.89 | 5.46 |
| Fujian | 28.44 | 0.80 | 175.96 | 4.95 | 409.43 | 11.52 |
| Guizhou | 19.41 | 0.06 | 116.73 | 0.37 | 329.45 | 1.03 |
| Shaanxi | 27.1 | 0.40 | 87.34 | 1.28 | 200.87 | 2.95 |
| Hainan | 14.99 | 0.23 | 70.90 | 1.09 | 164.89 | 2.53 |
| Shanghai | 14.06 | 0.54 | 65.75 | 2.53 | 139.55 | 5.37 |
| Beijing | 19.83 | 0.40 | 52.51 | 1.06 | 121.41 | 2.46 |
| Tianjin | 15.64 | 0.66 | 45.07 | 1.89 | 101.58 | 4.27 |
| Liaoning | 16.4 | 0.53 | 44.63 | 1.44 | 101.19 | 3.27 |
| Yunnan | 7.25 | 0.04 | 35.63 | 0.20 | 124.33 | 0.69 |
| Shanxi | 11.47 | 0.11 | 32.11 | 0.31 | 79.57 | 0.76 |
| Taiwan | 3.03 | 0.06 | 21.92 | 0.44 | 46.89 | 0.94 |
| Heilongjiang | 6.45 | 0.11 | 21.52 | 0.37 | 48.87 | 0.84 |
| Jilin | 7.48 | 0.19 | 20.71 | 0.52 | 44.41 | 1.12 |
| Hongkong | 2.76 | 0.11 | 18.44 | 0.71 | 33.77 | 1.30 |
| Nei Mongol | 4.15 | 0.10 | 11.62 | 0.28 | 29.34 | 0.70 |
| Gansu | 1.84 | 0.02 | 6.41 | 0.08 | 18.91 | 0.24 |
| Xinjiang | -0.46 | 0.00 | 6.40 | 0.05 | 18.75 | 0.14 |
| Ningxia | 1.47 | 0.02 | 4.83 | 0.06 | 13.31 | 0.16 |
| Macau | 0.35 | 0.01 | 2.07 | 0.08 | 3.99 | 0.15 |
| Tibet | 0.22 | 0.00 | 0.75 | 0.00 | 1.83 | 0.01 |
| Qinghai | 0.00 | 0.00 | 0.01 | 0.00 | 0.05 | 0.00 |

Economic Cost: billion USD. The 0.00 in and out of brackets represents the value is less than 0.001, but not equal to 0. Avoided WHL: the difference between annual WHL under the 1.5 °C scenario and three RCP scenarios.

**7. Future WHL by using different exposure-response functions**

As the Chinese exposure-response curves are slightly lower than the global curves under the common WBGT range (20°C ≤ WBGT ≤ 34°C) (Fig. S6-B), we found that the WHL estimated by the Chinese function under RCP2.6, RCP4.5, and RCP8.5 will be 4.5%, 3.4%, and 1.6% lower than that of the global function at the end of the century (Fig. S6). As part of the epidemiological evidence for the global ERF comes from tropical countries, it is reasonable for us to obtain a lower estimation.


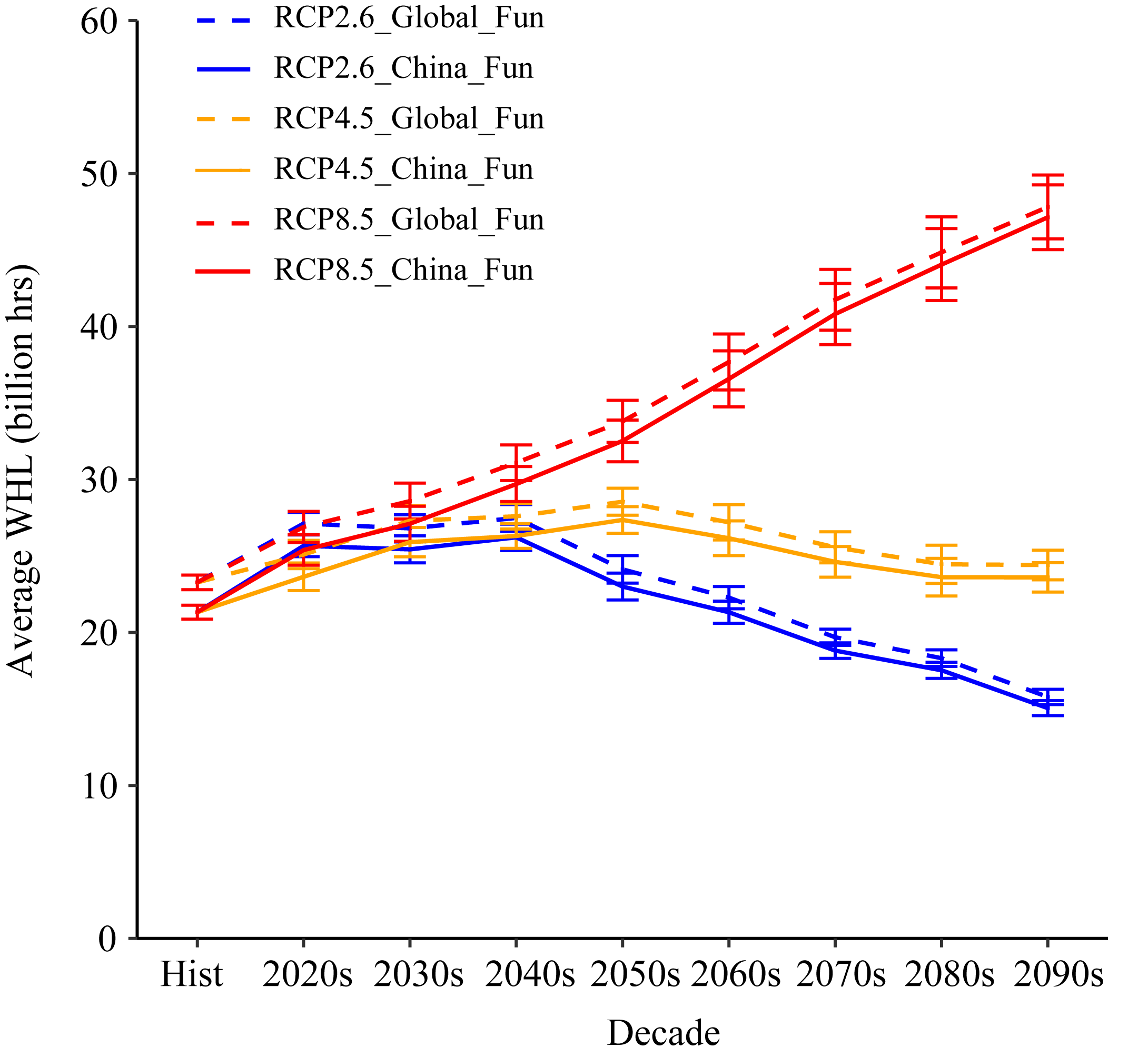


**Fig. S6.** Comparison of future heat-related WHL based on Chinese and global exposure-response functions. The solid lines represent the WHL estimated by the Chinese function. The dashed lines represent the WHL estimated by the global function. Fun: functions. Hrs: Hours.

**8. Future WHL under different scenarios of daily working hours**

Compared with the assumption of working 8 hours a day, the annual WHL of working 9 hours would increase by 0.4%, 1.1 %, and 2.5 %under the RCP 2.6, RCP 4.5 and RCP 8.5 scenarios at the end of this century, respectively. In addition, the annual average WHL of working 10 hours a day would increase by 0.9%, 2.2%, and 4.8% under the RCP 2.6, RCP 4.5 and RCP 8.5 scenarios at the end of this century, respectively. Since the time point of overtime is usually in the early evening, and the temperature is not as high as noon, the increase in WHL is not large.


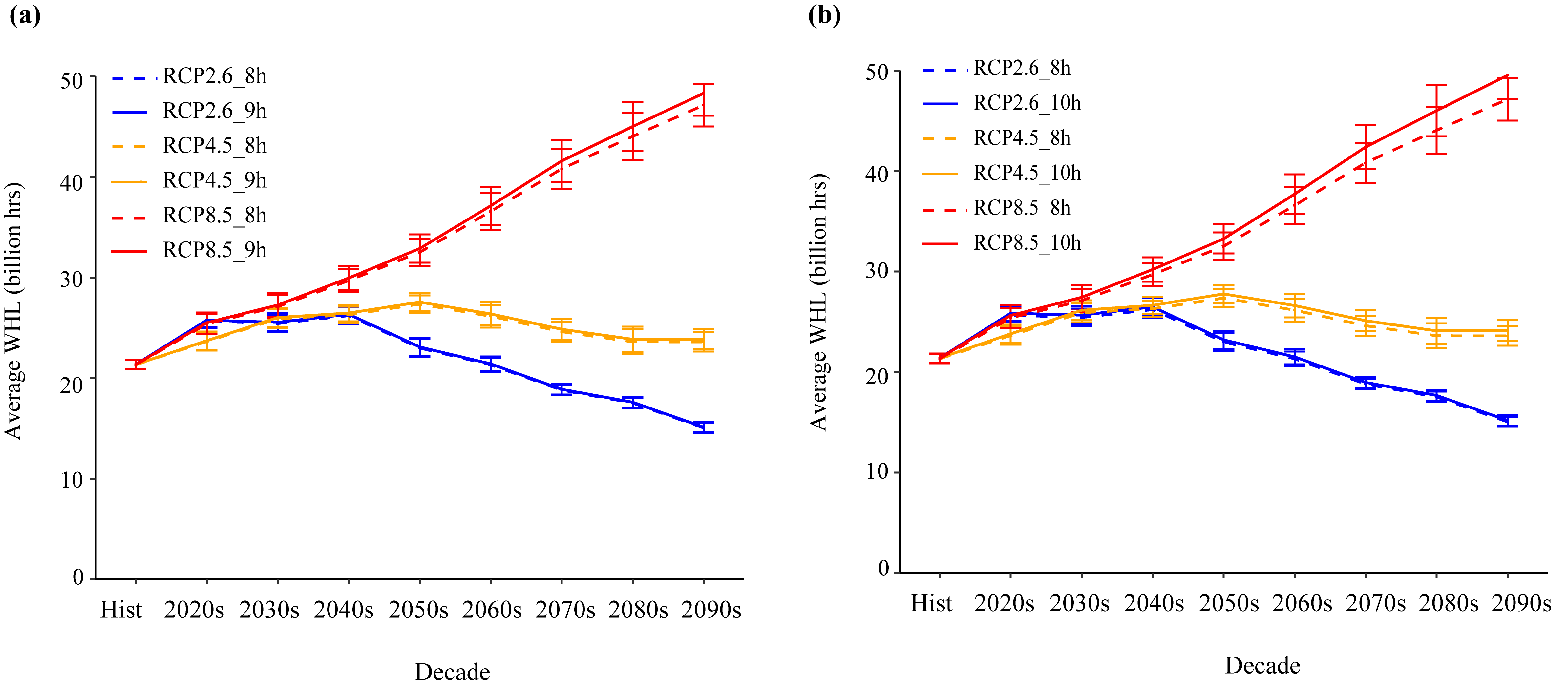


**Fig. S7.** Future heat-related WHL under the assumptions of different daily work hours. The solid lines represent the WHL under the assumption of working 9/10 hours a day. The dashed lines represent the WHL under the assumption of working 8 hours a day. Hrs: Hours.

**9. Future WHL under different scenarios of fertility rates**

Under the RCP2.6 scenario, the annual WHL under the high and low fertility scenarios are 9.2% higher and 12.3% lower than that in the medium fertility scenario at the end of the century, respectively. Under the RCP8.5 scenario, the annual WHL under the high and low fertility scenarios are 9.3% higher and 14.3% lower than the middle fertility scenario at the end of this century, respectively. Although there are corresponding increases and decreases in WHL in the high- and low-fertility scenarios, the overall trend remains the same (Fig. S8).

**
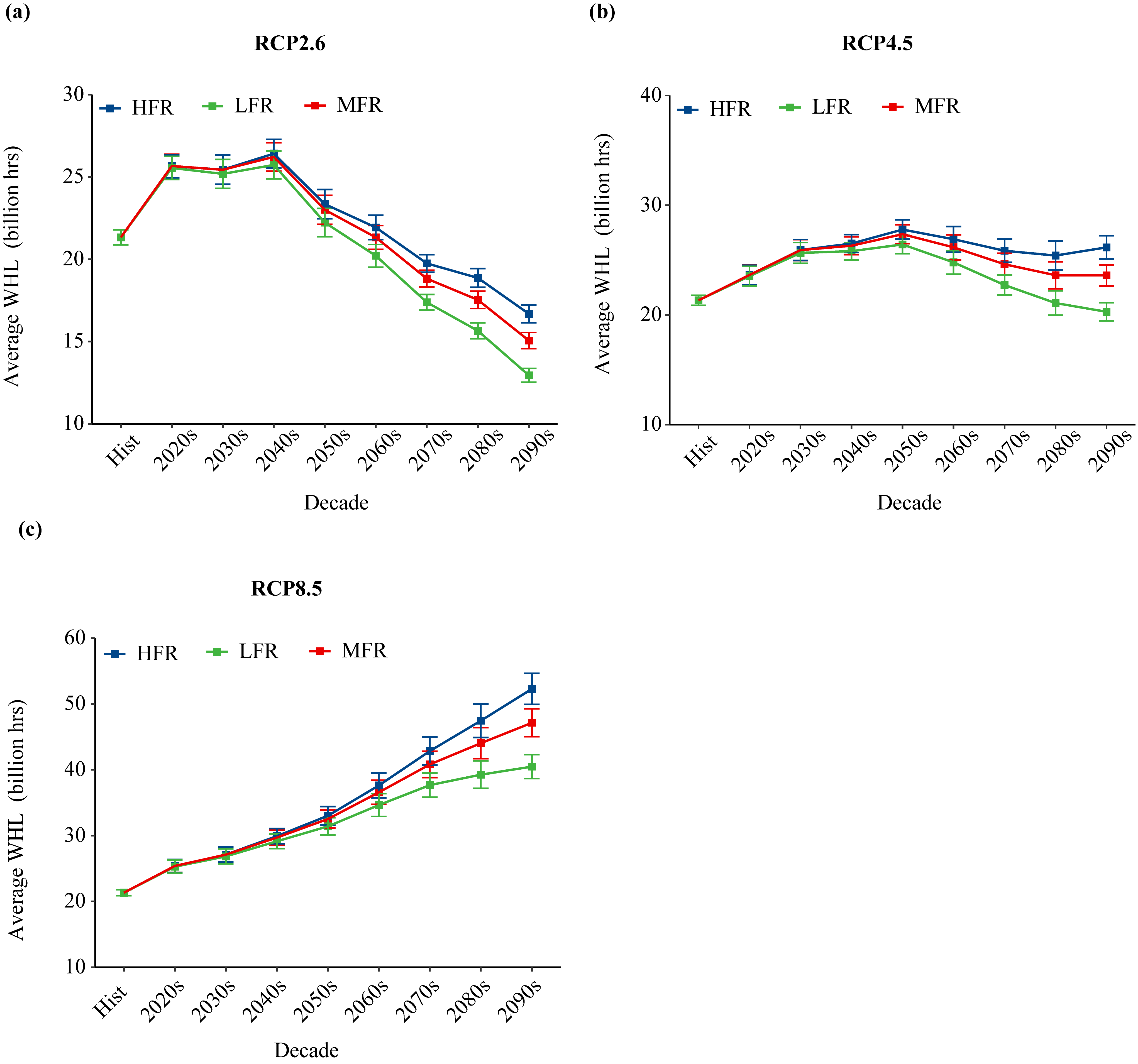
**

**Fig. S8.** Future heat-related WHL under scenarios with different fertility rates. LFR: low fertility rate. MFR: middle fertility rate. HFR: high fertility rate. Hist: baseline period in 1986-2005. Hrs: Hours.

**10. Future WHL under different scenarios of AC penetration rate**

If the future AC penetration rate will increase by 10% compared to the current rate, WHL will reduce by 6.7%, 7.3%, and 8.3% under RCP2.6, RCP4.5 and RCP8.5 at the end of the century, respectively. If the future AC penetration rate increases by 30% compared to the current, WHL will reduce by 20.0%, 21.9%, and 24.8% under RCP2.6, RCP4.5 and RCP8.5 at the end of the century, respectively (Fig. S9).


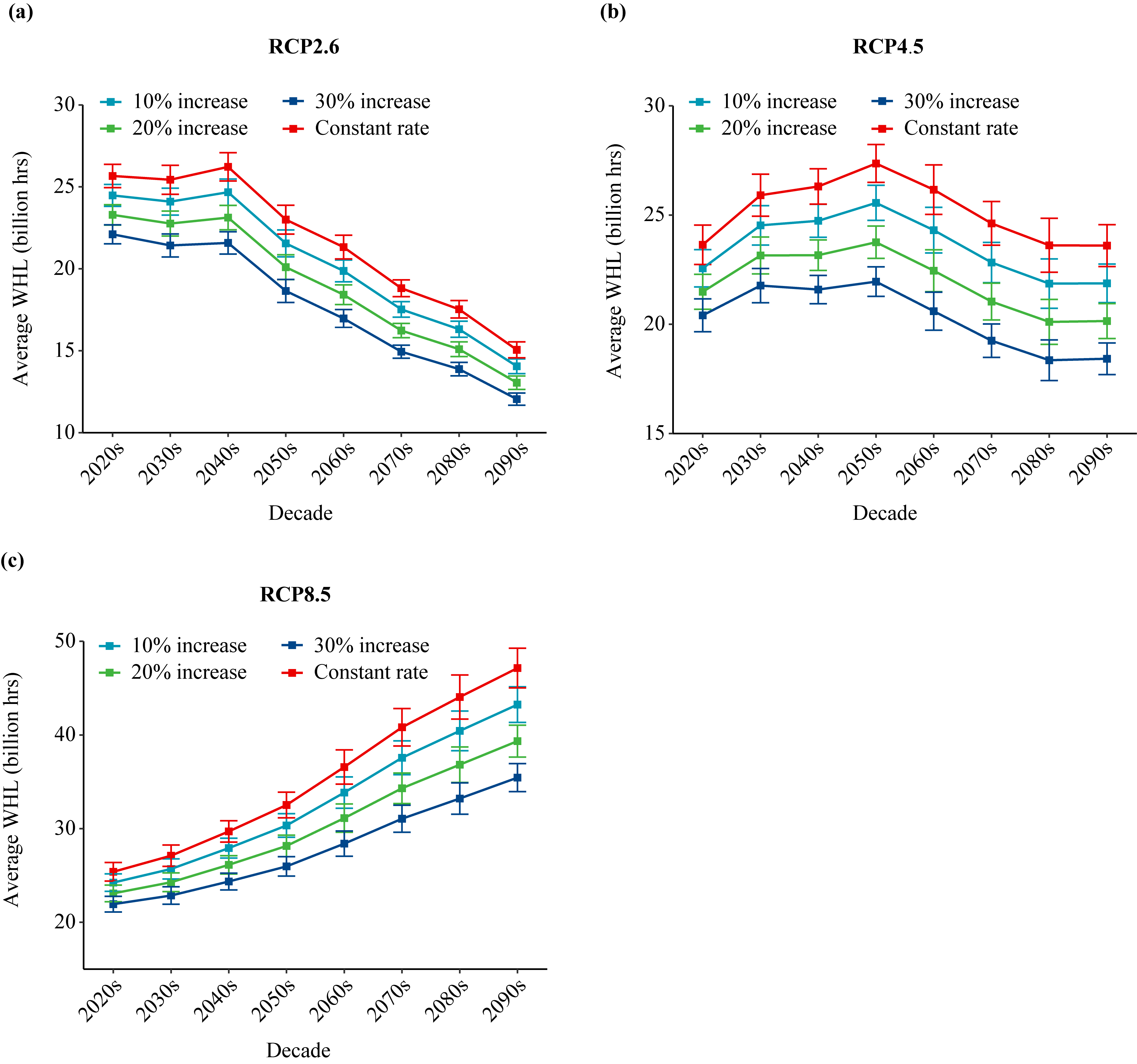


**Fig S9.** Future heat-related WHL under scenarios with different AC penetration rate. 10% increase, 20% increase and 30% increase denote compare to the present, the future average AC penetration rate will increase by 10%, 20%, and 30%, reaching 70%, 80%, and 90% by the end of the century, respectively.

**Supplementary References**

[1] Gao X, Shi Y, Giorgi F. Comparison of convective parameterizations in RegCM4 experiments over China with CLM as the land surface model. Atmos Ocean Sci Lett 2016;9:246-54.

[2] Gao X, Shi Y, Han Z, et al. Performance of RegCM4 over major river basins in China. Adv Atmos Sci 2017;34:441-55.

[3] Fu YH, Gao XJ, Zhu YM, et al. Climate change projection over the Tibetan Plateau based on a set of RCM simulations. Adv Clim Chang Res 2021;12:313-21.

[4] Giorgi F, Coppola E, Jacob D, et al. The CORDEX-CORE EXP-I Initiative: Description and highlight results from the initial analysis. Bull Amer Meteorol Soc 2022;103:E293-310.

[5] Yu E, Liu D, Yang J, et al. Future climate change for major agricultural zones in China as projected by CORDEX-EA-II, CMIP5 and CMIP6 ensembles. Atmos Res 2023;288:106731.

[6] Chen X, Guo Z, Zhou T, et al. Climate Sensitivity and Feedbacks of a New Coupled Model CAMS-CSM to Idealized CO2 Forcing: A Comparison with CMIP5 Models. J Meteorol Res-Prc 2019;33:31-45.

[7] Su B, Huang J, Fischer T, et al. Drought losses in China might double between the 1.5 °C and 2.0 °C warming. Proc Natl Acad Sci USA 2018;115:10600-5.

[8] Lemke B, Kjellstrom T. Calculating workplace WBGT from meteorological data: a tool for climate change assessment. Ind Health 2012;50:267-78.

[9] Liljegren JC, Carhart RA, Lawday P, et al. Modeling the wet bulb globe temperature using standard meteorological measurements. J Occup Environ Hyg 2008;5:645-55.

[10] Kjellstrom T, Freyberg C, Lemke B, et al. Estimating population heat exposure and impacts on working people in conjunction with climate change. Int J Biometeorol 2018;62:291-306.

[11] National Health Commission of China. Occupational exposure limits for hazardous agents in the workplace, Part 2: Physical agents. 2007. http://www.nhc.gov.cn/wjw/pyl/200705/39019.shtml (accessed Jan 8, 2022) (in Chinese).

[12] Cai W, Zhang C, Zhang S, et al. The 2021 China report of the Lancet Countdown on health and climate change: seizing the window of opportunity. Lancet Public Health 2021;6:e932-47.

[13] Yu S, Xia J, Yan Z, et al. Loss of work productivity in a warming world: Differences between developed and developing countries. J Clean Prod 2019;208:1219-25.

[14] Tong J, Buda S, Yanjun W, et al. Gridded datasets for population and economy under Shared Socioeconomic Pathways. Science Data Bank 2022. http://doi.org/10.57760/sciencedb.01683

[15] Huang J, Qin D, Jiang T, et al. Effect of Fertility Policy Changes on the Population Structure and Economy of China: From the Perspective of the Shared Socioeconomic Pathways. Earth's Future 2019;7:250-65.

[16] Jing C, Tao H, Jiang T, et al. Population, urbanization and economic scenarios over the Belt and Road region under the Shared Socioeconomic Pathways. J Geogr Sci 2020;30:68-84.

[17] He C, Zhang Y, Schneider A, et al. The inequality labor loss risk from future urban warming and adaptation strategies. Nat Commun 2022;13:3847.

[18] Orlov A, Sillmann J, Aunan K, et al. Economic costs of heat-induced reductions in worker productivity due to global warming. Glob Environ Change 2020;63:102087.

[19] Davis L, Gertler P, Jarvis S, et al. Air conditioning and global inequality. Glob Environ Change 2021;69:102299.

[20] Fatima SH, Rothmore P, Giles LC, et al. Extreme heat and occupational injuries in different climate zones: A systematic review and meta-analysis of epidemiological evidence. Environ Int 2021;148:106384.

[21] Broadbent AM, Krayenhoff ES, Georgescu M. The motley drivers of heat and cold exposure in 21st century US cities. Proc Natl Acad Sci USA 2020;117:21108-117.

[22] Zhao M, Lee JKW, Kjellstrom T, et al. Assessment of the economic impact of heat-related labor productivity loss: a systematic review. Clim Change 2021;167:22.

[23] Parsons LA, Masuda YJ, Kroeger T, et al. Global labor loss due to humid heat exposure underestimated for outdoor workers. Environ Res Lett 2022;17:014050.
