# Supplementary material for: Projecting future labor losses due to heat stress in China under climate change scenarios
Source: Sci Bull (Beijing). 2023 Nov 30;68(22):2827–37. doi: 10.1016/j.scib.2023.09.044 (PMC10694465; doi:10.1016/j.scib.2023.09.044)
Supplement: Supplementary data 2 [file mmc2.docx]

**气候变化情景下中国因高温造成的劳动生产力损失预估**

程亮亮^1,2^, 赵亮^3^, 谷魁英^2,13^, 王会滨^1^, 纪思翰^2^, 刘钊^4^, 黄建斌^5,6^, 陈艺丹^7^, 高学杰^8,9^, 徐影^10^, 王灿^7^, 罗勇^11^, 蔡闻佳^11^, 宫鹏^11,12^, 梁万年^2,13^, 黄存瑞^2,13,*^

^1^ School of Public Health, Sun Yat-sen University, Guangzhou 510080, China

^2^ Vanke School of Public Health, Tsinghua University, Beijing 100084, China

^3^ State Key Laboratory of Numerical modelling for Atmosphere Sciences and Geophysical Fluid Dynamics (LASG), Institute of Atmospheric Physics, Chinese Academy of Sciences, Beijing 100029, China

^4^ School of Linkong Economics and Management, Beijing Institute of Economics and Management, Beijing 100102, China

^5^ Beijing Yanshan Earth Critical Zone National Research Station, University of Chinese Academy of Sciences, Beijing 101408, China

^6^ College of Resources and Environment, University of Chinese Academy of Sciences, Beijing, 100190, China

^7^ State Key Joint Laboratory of Environment Simulation and Pollution Control (SKLESPC), School of Environment, Tsinghua University, Beijing 100084, China

^8^ College of Earth and Planetary Sciences, University of Chinese Academy of Sciences, Beijing 100084, China

^9^ Climate Change Research Centre, Institute of Atmospheric Physics, Chinese Academy of Sciences, Beijing 100029, China

^10^ National Climate Centre, China Meteorological Administration, Beijing 100081, China

^11^ Department of Earth System Science, Ministry of Education Key Laboratory for Earth System modeling, Institute for Global Change Studies, Tsinghua University, Beijing 100084, China

^12^ Department of Earth Sciences and Department of Geography, The University of Hong Kong, Hong Kong 999077, China

^13^ Institute of Healthy China, Tsinghua University, Beijing 100084, China

**摘要(350字左右)：**

气候变化背景下，高温会影响职业人群的工作效率并造成劳动生产力损失。本研究首次基于区域气候模式并采用本土化的暴露-反应函数，预估了未来气候变化情景下中国因高温造成的劳动时间损失。研究发现，与基线期的213亿小时高温相关劳动时间损失相比，到本世纪末在RCP2.6、RCP4.5和RCP8.5情景下，损失将分别增加-17.8%、10.8%及121.1%。损失主要集中在华南、华中和华东地区，其中广东与河南将占到全国总损失的四分之一，是因为这些区域的温度将更高、人口更密集、以及户外工作者在总人群中的比例较大。与RCP2.6、RCP4.5和RCP8.5情景相比，若能实现1.5°C温控目标，将分别能够避免11.8%、33.7%和53.9%的劳动时间损失，相当于避免了全中国本世纪中叶的0.1%、0.6%及1.4%的年均GDP损失。本研究显示未来气候变化会给中国造成严重的热相关劳动生产力损失，并强调亟需采取因地制宜的气候减缓及适应策略以保护职业人群健康。本研究对其它与中国相似的发展中国家同样具有重要意义。
